# Effectiveness of Chest Physiotherapy in Infants Hospitalized with Acute Bronchiolitis: A Multicenter, Randomized, Controlled Trial

**DOI:** 10.1371/journal.pmed.1000345

**Published:** 2010-09-28

**Authors:** Vincent Gajdos, Sandrine Katsahian, Nicole Beydon, Véronique Abadie, Loïc de Pontual, Sophie Larrar, Ralph Epaud, Bertrand Chevallier, Sylvain Bailleux, Alix Mollet-Boudjemline, Jean Bouyer, Sylvie Chevret, Philippe Labrune

**Affiliations:** 1 Assistance Publique – Hôpitaux de Paris (APHP), Pediatric Department, Hôpital Antoine Béclère, Clamart, France; 2 Inserm, CESP Centre for Research in Epidemiology and Population Health, U1018, Reproduction and Child Development Team, Villejuif, France; 3 Université Paris Sud 11, Paris, France; 4 APHP, Biostatistic Department, Hôpital Saint Louis, Paris, France; 5 Inserm UMRS U717, Paris, France; 6 APHP, Pulmonology Unit, Pediatric Department, Robert Debré Hospital, Paris, France; 7 Pediatric Department, Hôpital Necker Enfants Malades, Paris, France; 8 Université Paris 5, Paris, France; 9 APHP, Pediatric Department, Hôpital Jean Verdier, Bondy, France; 10 Université Paris Nord, Bobigny, France; 11 APHP, Pediatric Emergency Department, Necker Enfants Malades, Paris, France; 12 APHP, Pediatric Department, Hôpital Armand Trousseau, Paris, France; 13 Université Paris 6, Paris, France; 14 APHP, Pediatric Department, Hôpital Ambroise Paré, Boulogne, France; 15 Université Versailles, Saint Quentin en Yvelines, France; University of Liverpool, United Kingdom

## Abstract

**Background:**

Acute bronchiolitis treatment in children and infants is largely supportive, but chest physiotherapy is routinely performed in some countries. In France, national guidelines recommend a specific type of physiotherapy combining the increased exhalation technique (IET) and assisted cough (AC). Our objective was to evaluate the efficacy of chest physiotherapy (IET + AC) in previously healthy infants hospitalized for a first episode of acute bronchiolitis.

**Methods and Findings:**

We conducted a multicenter, randomized, outcome assessor-blind and parent-blind trial in seven French pediatric departments. We recruited 496 infants hospitalized for first-episode acute bronchiolitis between October 2004 and January 2008. Patients were randomly allocated to receive from physiotherapists three times a day, either IET + AC (intervention group, *n = *246) or nasal suction (NS, control group, *n = *250). Only physiotherapists were aware of the allocation group of the infant. The primary outcome was time to recovery, defined as 8 hours without oxygen supplementation associated with minimal or no chest recession, and ingesting more than two-thirds of daily food requirements. Secondary outcomes were intensive care unit admissions, artificial ventilation, antibiotic treatment, description of side effects during procedures, and parental perception of comfort. Statistical analysis was performed on an intent-to-treat basis. Median time to recovery was 2.31 days, (95% confidence interval [CI] 1.97–2.73) for the control group and 2.02 days (95% CI 1.96–2.34) for the intervention group, indicating no significant effect of physiotherapy (hazard ratio [HR]  = 1.09, 95% CI 0.91–1.31, *p = *0.33). No treatment by age interaction was found (*p = *0.97). Frequency of vomiting and transient respiratory destabilization was higher in the IET + AC group during the procedure (relative risk [RR]  = 10.2, 95% CI 1.3–78.8, *p = *0.005 and RR  = 5.4, 95% CI 1.6–18.4, *p = *0.002, respectively). No difference between groups in bradycardia with or without desaturation (RR  = 1.0, 95% CI 0.2–5.0, *p = *1.00 and RR  = 3.6, 95% CI 0.7–16.9, *p = *0.10, respectively) was found during the procedure. Parents reported that the procedure was more arduous in the group treated with IET (mean difference  = 0.88, 95% CI 0.33–1.44, *p = *0.002), whereas there was no difference regarding the assessment of the child's comfort between both groups (mean difference  = −0.07, 95% CI −0.53 to 0.38, *p = *0.40). No evidence of differences between groups in intensive care admission (RR  = 0.7, 95% CI 0.3–1.8, *p = *0.62), ventilatory support (RR  = 2.5, 95% CI 0.5–13.0, *p = *0.29), and antibiotic treatment (RR  = 1.0, 95% CI 0.7–1.3, *p = *1.00) was observed.

**Conclusions:**

IET + AC had no significant effect on time to recovery in this group of hospitalized infants with bronchiolitis. Additional studies are required to explore the effect of chest physiotherapy on ambulatory populations and for infants without a history of atopy.

**Trial registration:**

ClinicalTrials.gov NCT00125450

*Please see later in the article for the Editors' Summary*

## Abstract 2

Vincent Gajdos and colleagues report results of a randomized trial conducted among hospitalized infants with bronchiolitis. They show that a physiotherapy technique (increased exhalation and assisted cough) commonly used in France does not reduce time to recovery in this population.

## Editors' Summary

**Background:**

Bronchiolitis, which is usually caused by the respiratory syncytial virus (RSV), is the commonest infection of the lower respiratory tract (the lungs and the passages through which air enters the lungs) in infants. A third of all children have bronchiolitis during their first year of life. The illness begins with stuffiness, a runny nose, a mild cough, and mild fever. Then, as the smallest airways in the lung (the bronchioles) become inflamed (swell) and blocked with mucus, the cough worsens, and the infant may develop a wheeze, shallow breathing, and a rapid heartbeat. Most cases of bronchiolitis are mild and clear up within two weeks without any treatment but some infants develop severe disease. Such infants struggle to get enough air into their lungs, drawing in their chest with each breath (chest recession). They have trouble eating and drinking, and the oxygen level in their blood can drop dangerously low. About 1% of previously healthy infants need hospitalization because of severe bronchiolitis. These severely affected infants are not normally given any medications but, where necessary, they are given oxygen therapy, fed through a tube into their stomach, and given fluids through a vein.

**Why Was This Study Done?:**

In some countries, chest physiotherapy is routinely given to infants with bronchiolitis even though this is not a recommended treatment internationally. In France, for example, virtually all outpatients with bronchiolitis receive a form of chest physiotherapy known as increased exhalation technique with assisted cough (IET + AC). IET—manual chest compression—is designed to clear mucus from the bronchioles whereas AC—coughing triggered by applying pressure to the top of the breastbone—facilitates clearance of the large airways. But is IET + AC an effective treatment for bronchiolitis? In this study, the researchers undertook a multicenter, randomized, controlled trial to answer this question. A randomized trial is a study in which patients are randomly allocated to receive either the treatment under study or a control treatment. Usually in such trials, noone is aware of the treatment allocations until the trial has been completed. This is called blinding and avoids unconscious biases being introduced into the results. In this trial, although the parents, caregivers, and outcome assessors were blinded, the physiotherapists and the infants were aware of treatment allocations. The physiotherapists were not involved in patient assessment, however, and the infants were sufficiently young that their knowledge of their treatment was unlikely to bias the results.

**What Did the Researchers Do and Find?:**

The researchers enrolled nearly 500 children aged 15 days to 2 years who were admitted to seven French hospitals for a first episode of acute bronchiolitis. They randomly allocated the patients to receive IET + AC (intervention group) or nasal suction (control group) three times a day from a physiotherapist working alone in a room with blacked-out windows. The primary outcome of the trial was the patients' time to recovery. Infants were judged to have recovered if they had not had oxygen therapy or showed signs of chest recession for 8 hours and had ingested more than two-thirds of their daily food requirement. Infants in the control group took an average of 2.31 days to recover whereas those in the intervention group took 2.02 days. However, this difference in recovery time was not statistically significant. That is, it could have happened by chance. The researchers also recorded several secondary outcomes such as admission to an intensive care unit, help with breathing, antibiotic treatment, and parental perceptions of their child's comfort. There were no significant differences between the two treatment groups for any of these secondary outcomes, although the parents did report that the IET + AC treatment was harder on their children than nasal suction while not reducing their overall comfort.

**What Do These Findings Mean?:**

These findings show that IET + AC had no significant effect on the time to recovery of a large population of French infants admitted to hospital with severe bronchiolitis. These results cannot be extrapolated, however, to infants with mild or moderate bronchiolitis, and further studies are needed to assess whether chest physiotherapy is of any benefit in an outpatient setting. Three small trials of a different form of chest physiotherapy have also previously failed to find any effect of chest physiotherapy on recovery time. Thus, none of the currently available results support the routine use of chest physiotherapy in infants admitted to a hospital for severe bronchiolitis.

**Additional Information:**

Please access these Web sites via the online version of this summary at http://dx.doi.org/10.1371/journal.pmed.1000345

- The UK National Health Service Choices Web site provides detailed information on all aspects of bronchiolitis
- Kidshealth, a resource maintained by the Nemours Foundation (a not-for-profit organization for children's health) provides information for parents on bronchiolitis schizophrenia and on respiratory syncytial virus (in English and Spanish)
- The British Lung Foundation also provides information on bronchiolitis schizophrenia and on respiratory syncytial virus
- The MedlinePlus encyclopedia has a page on bronchiolitis schizophrenia (in English and Spanish)
- The US Centers for Disease Control and Prevention has detailed information on respiratory syncytial virus

## Introduction

Acute viral bronchiolitis is generally a self-limiting condition and is most commonly associated with respiratory syncytial virus (RSV) infection. It is the most common lower respiratory tract infection in the first years of life, and it is usually a mild to moderate disease. However, some infants develop severe disease and hospitalization is necessary in about 1% of previously healthy infected infants [1]–[3]. Recent population-based studies have shown that mean annual hospitalization rates for conditions related to RSV infection were between 5 and 17 per 1,000 children under 12 mo of age [4],[5].

The treatment of bronchiolitis in infants is largely supportive, with oxygen supplementation, minimal handling of the infant, and the use of enteral feeding, intravenous fluids, or ventilatory support when necessary. Respiratory symptoms result from bronchiolar obstruction due to inflammatory edema in the small airways, with an accumulation of mucus and cellular debris secondary to the epithelial necrosis caused by the virus [6]–[8].

Chest physiotherapy is routinely used in children with chronic respiratory diseases, such as cystic fibrosis and primary ciliary dyskinesia, or in children with neuromuscular disease, to facilitate the clearance of tracheobronchial secretions. Chest physiotherapy aims to clear airway obstruction, thereby decreasing airway resistance, improving gas exchange, and making breathing easier. International recommendations do not recommend chest physiotherapy for the management of bronchiolitis [9],[10]. These recommendations are based on a recent Cochrane review analyzing three clinical trials evaluating chest physiotherapy in infants with bronchiolitis [11]; however, these studies have several limitations. Most importantly, these studies were based on percussion and vibration techniques, which are very different from the increased exhalation technique (IET) with assisted cough (AC) that might be more appropriate in this context. IET is designed to clear the distal airways, whereas AC is known to facilitate large-airway clearance [12],[13]. During IET, the manual compression of the infant's thorax is aimed at achieving distal airways flow limitation at low lung volume (as during the rapid thoracic compression lung function technique [14]–[16]) to facilitate mucus clearance.

A French consensus panel recommended the use of IET + AC in infants with bronchiolitis [17]. Despite the very large use of IET + AC in outpatients with bronchiolitis in France (82.5%–99%) [18], the efficacy of this technique has so far not been evaluated.

Thus, we conducted a multicenter, randomized, outcomes assessor-blind and parent-blind trial to evaluate the efficacy of chest physiotherapy by IET + AC in previously healthy infants, hospitalized for a first episode of acute bronchiolitis.

## Methods

### Ethics Statement

The Saint Germain en Laye ethics committee approved the study.

### Patients

We conducted the study in seven French pediatrics departments in the Parisian area (Antoine Béclère, Clamart; Jean Verdier, Bondy; Ambroise Paré, Boulogne; Kremlin Bicêtre, Le Kremlin Bicêtre; and Paris, Robert Debré, Necker Enfants Malades, and Armand Trousseau) during bronchiolitis outbreaks from October 2004 through January 2008 (Texts S1 and S2). Planned start and end dates were identical for all centers.

We included infants between the ages of 15 d and 24 mo hospitalized with a first episode of wheezing diagnosed as bronchiolitis. Bronchiolitis was diagnosed on the basis of a history of upper respiratory tract infection and clinical findings consistent with bronchiolitis, including wheezing or wheezing with crackles and respiratory distress. Infants were eligible within 24 h of hospitalization if they presented at least one of the following on admission: toxic aspect; history of apnea or cyanosis; respiratory rate >60/min; pulse oxymetry <95%; alimentary intake <2/3 of needs. A maximum of two chest physiotherapy procedures since admission was allowed before inclusion.

Infants with severe respiratory distress necessitating immediately admission to the pediatric intensive care unit (PICU), with cardiac disease, with a previous significant respiratory condition, or premature (<34 wk) were not eligible. Infants were not included if they had contraindications for the intervention (IET + AC): thrombocytopenia, prolonged corticosteroid treatment, rickets, bone diseases, known rib fracture.

The parents were informed about the study, its aims, and design. In particular, they were informed that they could not stay with their children during treatment (to respect blinding). Both parents gave written informed consent.

Clinical bronchiolitis was confirmed at enrollment and clinicians determined the duration of symptoms before hospitalization, clinical respiratory score, and clinical variables (respiratory and heart rates, temperature, and oxygen saturation whilst breathing ambient air). Medical history was obtained from parents or guardians, on a standardized data-collection form including questions about personal history of eczema, family history of asthma or eczema in parents and siblings, and tobacco smoke in the home environment.

### Randomization

Randomization involved the chest physiotherapist opening a sealed sequentially numbered envelope containing a random allocation computer generated with SAS (SAS Inc.) software packages in advance by the biostatistician. Randomization was stratified according to center and according to age (<2 mo, ≥2 mo) at each center, using permutation blocks with a block size of four that was not mentioned to the physicians involved in the patient recruitment.

All pediatric department staff, parents, and guardians were blind to treatment assignment. Randomization codes were kept secure until data entry was complete. Thus, those involved in the evaluation of primary outcome or in the decision of the cointerventions were blinded to group assignment.

### Study Intervention

The treatment, either intervention or control, was performed by the physiotherapist staying alone with the infant, in a room with a covered window pane, to ensure that clinicians and parents could not observe treatment, thus preserving blinding in the trial. All infants received treatment three times daily. In each center, four to six physiotherapists, specially trained to carry out chest physiotherapy in children, participated in the study. The therapists were not involved in the evaluation of time to recovery.

#### Intervention group

The intervention was defined as the IET followed by AC, with gentle nasal suction (NS).

Just before the start of the study, a senior physiotherapist presented this technique at each center and all physiotherapists received formal training in these techniques. During the study, a referent physiotherapist at each center ensured that chest physiotherapy was consistent and standardized.

IET involved the generation of synchronized thoracic-abdominal movement by the hands of the physiotherapist at the beginning of expiration with one hand on the thorax, meanwhile, with the other on the abdomen, centered on the umbilicus, the physiotherapist applied an abdominal counter-weight. This maneuver allowed to create a passive expiratory flow. The maneuver began at the end of the inspiratory plateau and was pursued until the end of expiration, according to the infant's thoraco-pulmonary compliance and up to his or her chest wall and lung resistance limits. The resulting dynamic compression of the respiratory system increased expiratory airflow. The procedure was repeated until meeting auscultation-efficacy criteria (decrease or disappearance of wheezing and/or increase of ronchi), but did not last longer than 10 to 15 min. The procedure was stopped in the case of respiratory status aggravation. If no spontaneous coughing occurred, coughing could be triggered by pressure on the suprasternal notch. Gentle NS with a flexible probe was used to remove mucous secretions at the end of the procedure. All patients were closely monitored by continuous pulse oxymetry during chest physiotherapy.

#### Control group

Similarly to the intervention group, the control group also spent 10–15 min in a room alone with the therapist three times daily. In this group of infants, the physiotherapists performed only gentle NS to remove mucous secretions for few minutes and stayed inside the room for the remaining time without performing any maneuver on the infants.

### Other Treatments

All children enrolled in this study followed the same clinical treatment pathway to ensure consistent care and minimal variability of the results. Guidelines for the use and termination of oxygen supplementation and orogastric feeding or intravenous fluids were followed.

Oxygen supplementation was administered if oxygen saturation was below 95% when awake and 92% when asleep. It was stopped when oxygen saturation was consistently above 95% when awake and 92% when asleep. Nurses interrupted oxygen supplementation three times per day to assess saturation in room air.

Enteral feeding was administered when possible, with orogastric feeding offered to infants spontaneously ingesting less than two-thirds of their daily needs or with significant signs of chest recession, tachypnea (>60/min) or hypoxemia, or a worsening of respiratory signs during feeding. Intravenous fluids were preferred over oral feeding if respiratory conditions did not improve with orogastric feeding or oral feeding was insufficient (particularly if vomiting occurred during orogastric feeding). The use of intravenous fluids stopped when the infant was able to tolerate oral feeding. A physician and nurses reevaluated the need for orogastric feeding and intravenous fluids twice daily.

Other treatments, such as bronchodilators, corticosteroids, and antibiotics, are not recommended in national and international guidelines [2],[3],[10],[19], but could be prescribed freely if physicians felt it appropriate. Data concerning deviations from the clinical treatment pathway, including drug treatments, were recorded.

### Outcome Measure

#### Primary outcome

The primary outcome was time from randomization to recovery. An infant was considered to be cured if no oxygen supplementation had been given for 8 h and the child had minimal or no chest recession and was ingesting more than two-thirds of daily needs. The nursing staff recorded respiratory and heart rates, oxygen saturation, and signs of chest recession when the patient was quiet, at least once every 8 h. Evaluation was based on a clinical score that could be recorded reliably, every 8 h, by any doctor, nurse, or physiotherapist [20].

#### Secondary outcomes

First, physiotherapists reported side effects during procedures: bradycardia (<80/min) without desaturation, bradycardia with desaturation (SpO_2_<85%), vomiting, transient respiratory destabilization, or bouts of hypotonia requiring the interruption of the procedure.

Upon discharge from the hospital, parents answered a questionnaire regarding their perception of their child's comfort and were invited to give their opinion on the efficiency of physiotherapy for their own child. As parents were not allowed to be present during the physiotherapy procedure, their opinion was a global one, based on their observations before and after each procedure, and on their global evaluation of their child. Parents answered several questions: (1) During hospitalization, how would you evaluate the comfort of your child? Analogical visual scale ranging from 0 (very bad) to 10 (excellent); (2) How would you evaluate how arduous the physiotherapy procedure was for your child? Analogical visual scale ranging from 0 (not arduous at all) to 10 (very arduous); (3) Do you think that the physiotherapy procedure has really worsened, rather worsened, not changed anything, rather improved, or really improved the comfort of your child (choose one answer)?; and (4) Do you think that the physiotherapy procedure has really worsened, rather worsened, not changed anything, rather improved, or really improved your child's breathing (choose one answer).

Finally, secondary PICU admission and artificial ventilation, antibiotic treatment were recorded. The parents were contacted by telephone within 30 d of discharge to identify cases of relapse and rehospitalization.

### Computation of Sample Size

No accurate data for mean time to recovery were available from the literature. Therefore, to determine the sample size, we used the duration of hospitalization for bronchiolitis recorded in the study hospitals during previous years (mean duration of hospitalization, 6.5 d, and standard deviation [SD], 3.5 d). For a type I error of 0.05 and a power of 0.80, for detecting a 20% decrease in time to recovery in the IET + AC group, we needed to include 228 infants (114 infants in each group). One of the aims of this trial was to investigate possible interactions with age. We therefore set up two groups of 228 children (under and over the age of 2 mo), giving 456 children in total. We planned to include an additional 10% of patients to ensure that we had sufficient subjects for analysis (due to potential study dropouts or consent withdrawals). We therefore planned to enrol 500 infants in this trial.

### Statistical Analysis

Analysis was performed on an intent-to-treat basis and all patients included in the study were analyzed, including the two lost to follow-up (one in each group).

Baseline demographic data were expressed as number and percentage for binary or ordinary data, and means ±SD for continuous data unless skewed, where median and interquartile range (IQR) were reported. Time failure data were summarized as medians and 95% confidence interval (CI) [21].

We first tested treatment by age group (<2 mo and ≥2 mo) interaction on the primary outcome by fitting Cox models in each age group, then testing for quantitative interaction with the Gail and Simon test [22]. No treatment by age interaction was found (*p = *0.97), making it possible to perform the analysis on the pooled sample. Thus, survival curves for time to recovery were estimated on the whole cohort using the Kaplan-Meier method, then compared across randomized groups by using the log-rank test stratified by age group.

We additionally adjusted survival analyses for prognostic baseline covariates (personal eczema or history of atopy, age in months, hypoxemia at randomization, need for IV fluids at randomization, atelectasia at randomization, duration of symptoms, use of mucolytics before randomization, RSV infection), using a Cox model. The center effect, that is, difference in baseline risk between centers, was analyzed using frailty models [23].

For secondary outcomes, we compared adverse events frequency using the Fisher test. The need for PICU admission or ventilation, lung atelectasia, relapse, and the need for antibiotic treatment or secondary hospitalization were compared between the two groups using the chi square test stratified on age. Data from analogical visual scales were compared using Wilcoxon test.

Finally, we tested treatment by covariate interactions on the primary outcome with personal eczema or history of atopy (history of atopy was defined as eczema or asthma in first-degree relatives), hypoxemia (SpO_2_<95%) at randomization, and RSV infection. These analyses were not prespecified and were identified by post hoc analysis. All these quantitative interactions were tested with the Gail and Simon test [22].

Measures of treatment effect were either hazard ratio (HR) for survival data, relative risk (RR) for binary data, mean differences for continuous data, all given with 95% CIs. For continuous skewed variables, 95% CIs were obtained by the bootstrap method [24]. All tests were two-tailed, with *p*-values of 0.05 or less considered as statistically significant.

Statistical analysis was carried out with R version 2.10.11 (The R Foundation for Statistical Computing; http://www.R-project.org) and SAS version 9.2 (SAS Inc.).

## Results

### Patients and Baseline Characteristics

**Figure 1:**
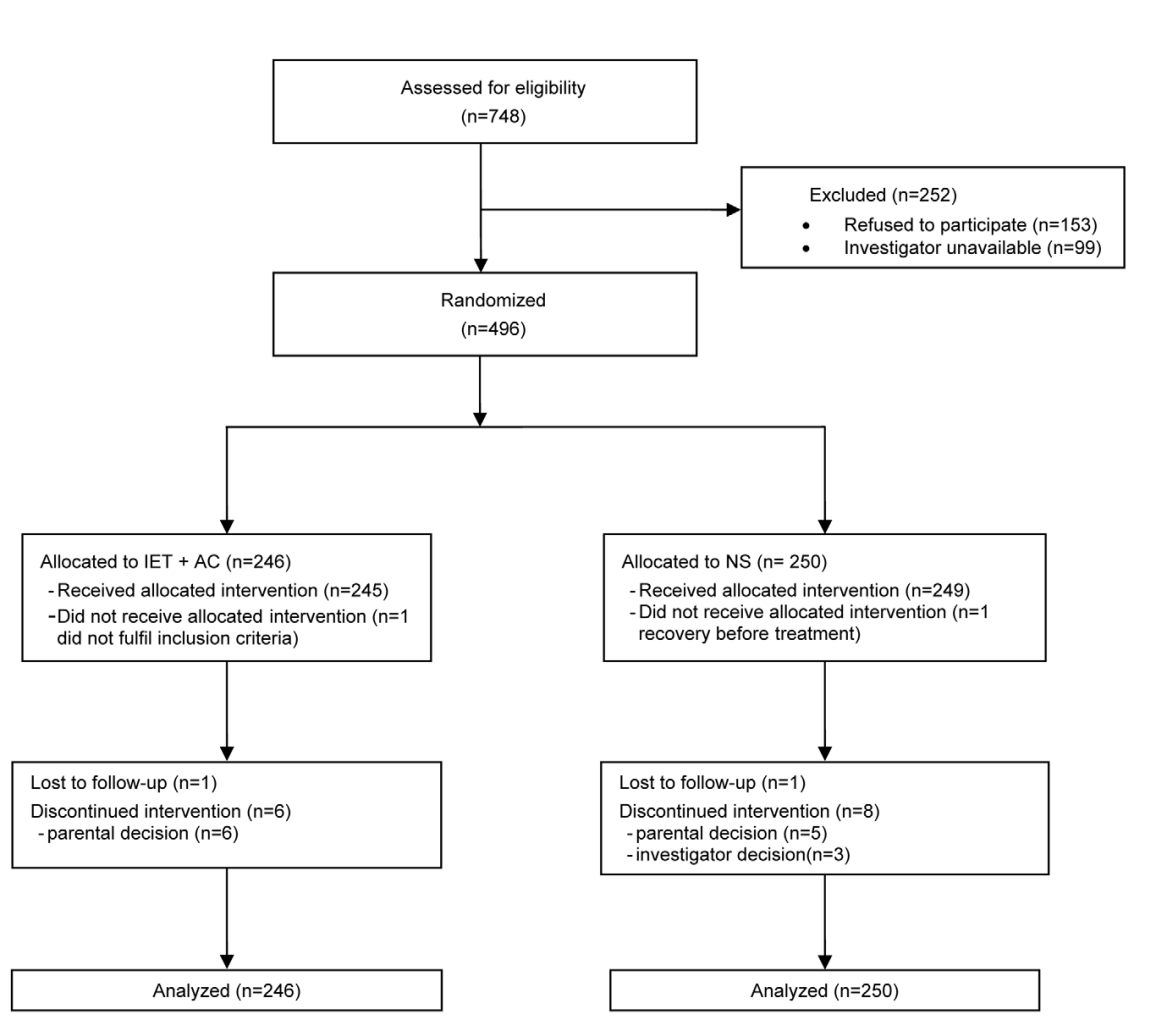
Eligibility, randomization, and follow-up of study participants.

496 infants were included from October 2004 to January 2008 (Figure 1): 246 (49.6%) were assigned to the IET + AC group and 250 (50.4%) to the NS group. For153 additional infants whose parents were invited to participate, the parents refused participation. The reasons for their refusal were the desire to stay with their infant, and therefore the impossibility to maintain the blind, and the desire for their children to receive IEC + AC treatment.

At randomization, there were no marked differences between the two randomized groups for demographic variables, percentage of infants with hypoxemia or feeding difficulties, percentage of infants with nasal aspirate positive for RSV, and duration of respiratory symptoms before hospitalization (Table 1). The proportion of cases of lung atelectasia diagnosis on X-ray was higher in the NS group (12.9% versus 7.6%).

**Table 1:** Demographic characteristics of the infants on admission to the hospital.

Characteristic	NS (*n = *250)	IET + AC (*n = *246)
**Age (mo), median [IQR]**	2.0 [1.3–4.0]	2.1 [1.3–3.8]
**Male gender, ** ***n*** ** (%)**	141 (56.4)	134 (54.5)
**Gestation (wk), mean ± SD**	39.1±1.65	39.1±1.67
**Environmental tobacco smoke** a **, ** ***n*** ** (%)**	69 (29.0)	65 (26.9)
**Personal eczema or history of atopy** a,b **, ** ***n*** ** (%)**	100 (40.7)	97 (39.8)
**Kindergarten** a **, ** ***n*** ** (%)**	37 (15.0)	29 (12.0)
**Bronchodilators before randomization** a **, ** ***n*** ** (%)**	36 (14.4)	47 (19.1)
**Corticosteroids before randomization** a **, ** ***n*** ** (%)**	34 (13.6)	25 (10.2)
**Feeding difficulties before randomization** a **, ** ***n*** ** (%)**	222 (89.2)	207 (84.8)
**Duration of respiratory symptoms at randomization in days** a **, median [IQR]**	3.0 [2.0–4.0]	3.0 [2.0–4.0]
**SpO_2_<95% at randomization, ** ***n*** ** (%)**	110 (44.2)	106 (44.2)
**Atelectasia at randomization** c **, n (%)**	31 (12.9)	18 (7.6)
**Supplementary oxygen and intravenous feeding, ** ***n*** ** (%)**		
** No oxygen**	141 (56.4)	135 (54.9)
** Oxygen only**	86 (34.4)	86 (34.9)
** Oxygen and intravenous feeding**	23 (9.2)	25 (10.2)
**RSV +, ** ***n*** ** (%)**	152 (76.4)	137 (73.3)
**Temperature at randomization (°C), mean ± SD**	37.2±0.7	37.3±0.6

a The data were obtained by parental reporting.

b History of atopy was defined as eczema or asthma in first-degree relatives.

c 480 (96.7%) had X-ray at admission.

Before randomization, 36 (14.4%) infants in the NS group and 47 (19.1%) infants in the IET + AC group received bronchodilator inhalations (salbutamol). 34 (13.6%) infants in the NS group and 25 (10.2%) infants in the IET + AC group were treated with oral corticosteroids (betamethasone).

### Primary End Point

**Figure 2:**
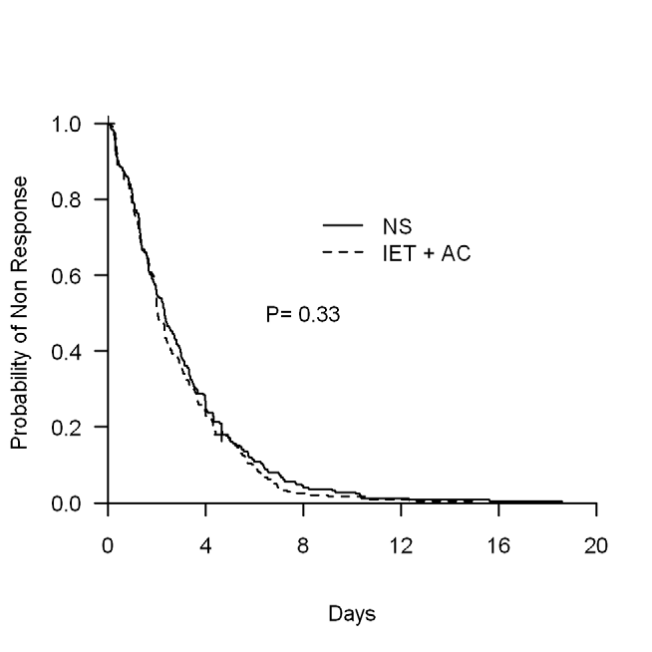
Kaplan-Meier estimates of the proportion of children not recovering.

The physiotherapy intervention (IET + AC) had no significant effect on time to recovery. The median (95% CI) time to recovery was 2.31 (1.97–2.73) d for the NS group and 2.02 (1.96–2.34) d for the IET + AC group (HR [95% CI]  = 1.09, 0.91–1.31, *p = *0.33; Figure 2; Table 2). Significant heterogeneity across centers in baseline hazards was observed (*p = *0.001), reflecting a difference between centers in time to recovery independently of the treatment effect. After controlling for prognostic baseline covariates, the random effect Cox regression model, that takes into account the center effect, showed that the effect of IET + AC on time to recovery remained not significant (HR  = 1.21, 0.97–1.49, *p = *0.09). Of note, the center effect persisted, though erased by the handling of baseline prognostic covariate differences (*p = *0.03).

**Table 2:** Estimated effect of increased exhalation technique on time to recovery (d) as a function of age (univariate analyses).

Population	NS	IET + AC	HR [95% CI] of recovery	*p*-Value*
	Median time to recovery, d [95% CI]			
**Overall (** ***n = *** **496)**	2.31 [1.97–2.73]	2.02 [1.96–2.34]	1.09 [0.91–1.31]	0.33
**<2 mo (** ***n = *** **238)**	2.64 [2.25–3.08]	2.47 [1.98–3.31]	1.09 [0.84–1.41]	0.51
**≥2 mo (** ***n = *** **258)**	2.01 [1.65–2.44]	2.00 [1.51–2.25]	1.09 [0.85–1.40]	0.48

*Log-rank test (stratified by age group for overall analysis).

### Secondary End Points

Table 3 summarizes side effects during procedures reported by physiotherapists in both groups: there were no significant difference between groups in the proportion of children who experienced one episode of bradycardia with desaturation (RR [95% CI]  = 1.0, 0.2–5.0, *p = *1.00) or without desaturation (RR  = 3.6, 0.7–16.9, *p = *0.10). Conversely, the proportion of children who either had transient respiratory destabilization or vomited during the procedure was significantly higher in the group treated with IET + AC than in the group treated with NS (RR  = 10.2, 1.3–78.8, *p = *0.005 and RR  = 5.4, 1.6–18.4, *p = *0.002, respectively).

**Table 3:** Side effects reported by physiotherapists during procedures.

Side Effect	NS (*n = *250)	IET + AC (*n = *246)	Relative Risk [95% CI]	*p*-Value^a^
**Bradycardia with desaturation, ** ***n*** ** (%)**	3 (1.2%)	3 (1.2%)	1.0 [0.2–5.0]	1.00
**Bradycardia without desaturation, ** ***n*** ** (%)**	2 (0.8%)	7 (2.8%)	3.6 [0.7–16.9]	0.10
**Vomiting during procedure**	1 (0.4%)	10 (4.1%)	10.2 [1.3–78.8]	0.005
**Respiratory destabilization**	3 (1.2%)	16 (6.5%)	5.4 [1.6–18.4]	0.002
**Hypotonia**	0 (0.0%)	2 (0.8%)	NA	0.24

a Fischer exact test.

We obtained 371 (74.8%) responses to parental questionnaire (187 in the NS group and 184 in the IET + AC group). Table 4 summarizes parental answers regarding the influence of the physiotherapist visit on both the child's comfort and respiratory status. According to the parents, the procedure was significantly more arduous in the group treated with IET (mean difference [95% CI]  = 0.88, 0.33–1.44, *p = *0.002), whereas there was no significant difference regarding the comfort of the child between both groups (Difference of mean  = −0.07, −0.53 to 0.38, *p = *0.40). Finally, there was no significant difference in the way the parents rated the influence of physiotherapy on respiratory status (RR  = 0.99, 0.90–1.08, *p = *0.89 or comfort (RR = 0.99, 0.94–1.05, *p = *0.84).

**Table 4:** Parental opinions regarding the comfort of their child and the consequences of the procedure on this parameter and on the respiratory status.

Parental Opinion	NS (*n = *187)	IET + AC (*n = *184)	Mean Difference [95% CI]	Relative Risk [95% CI]	*p*-Value
**Evaluation de the comfort of your child during hospitalization, median (IQR)**	7.8 [5.7–9.0]	7.5 [6.2–8.7]	−0.07 [−0.53 to 0.38]	—	0.40^a^
**Evaluation of the procedure arduous, median (IQR)**	4.3 [2.0–6.3]	5.0 [3.0–7.1]	0.88 [0.33–1.44]	—	0.002^a^
**Influence of the physiotherapist visit on the comfort of your baby, ** ***n*** ** (%)**			—	0.99 [0.90–1.08]^b^	0.89^c^
Worsening	5 (2.7%)	12 (6.5%)	—	—	—
No influence	25 (13.4%)	19 (10.3%)	—	—	—
Improvement	157 (83.9%)	153 (83.2%)	—	—	—
**Influence of the physiotherapist visit on the respiratory status of your baby, ** ***n*** ** (%)**			—	0.99 [0.94–1.05]^b^	0.84^c^
Worsening	4 (2.1%)	1 (0.5%)	—	—	—
No influence	8 (4.3%)	12 (6.6%)	—	—	—
Improvement	175 (93.6%)	170 (92.9%)	—	—	—

371 couples of parents (74.8%) completed the questionnaire. Percentages were calculated for the population of respondents (*n = *371).

a Wilcoxon test.

b Relative risk was computed for improvement versus (no influence + worsening).

c Fischer Exact test.

17 (3.4%) infants, including seven (1.4%) requiring ventilatory support, were admitted to the PICU. There was no evidence of any difference between groups in the number of infants requiring intensive care admission (RR = 0.7, 0.3–1.8, *p = *0.62) or ventilatory support (RR = 2.5, 0.5–13.0, *p = *0.29). 69 (28.5%) infants in the NS group and 67 (28.6%) in the IET + AC group received antibiotics (RR = 1.0, 0.7–1.3, 1.00). 53 infants in each group (31.0% in the IET + AC group) relapsed within 1 mo after discharge (RR = 1.1, 0.8–1.5, *p = *0.73). 26 patients were admitted to the hospital within 1 mo after discharge (12 in the NS group and 14 in the IET + AC group; RR = 1.2, 0.6–2.6, *p = *0.68) (Table 5). Respiratory symptoms were responsible for rehospitalization in 16 (six in the NS group and ten in the IET + AC group) out of 26 cases. Gastroenteritis was the other main cause (seven out of 26) of secondary hospitalization; the three remaining infants were hospitalized for miscellaneous nonrespiratory disorders.

**Table 5:** Secondary outcomes.

Secondary Outcome	NS (*n = *250)	IET + AC (*n = *246)	Relative Risk [95% CI]	*p*-Value a
**PICU admission, ** ***n*** ** (%)**	10 (4.1%)	7 (2.9%)	0.7 [0.3–1.8]	0.62
**Ventilation, ** ***n*** ** (%)**	2 (0.8%)	5 (2.0%)	2.5 [0.5–13.0]	0.29
**Antibiotics**	69 (28.5%)	67 (28.6%)	1.0 [0.7–1.3]	1.0
**Relapse**	53/182 (29.1%)	53/171 (31.0%)	1.1 [0.8–1.5]	0.73
**New hospitalization**	12/182 (6.6%)	14/171 (8.2%)	1.2 [0.6–2.6]	0.68

Data obtained from the parents by telephone interview 30 d after discharge. We obtained 353 responses (71.2%) and percentages were calculated for the population of respondents (*n = *353).

a Fischer exact test for percentage comparison.

### Post Hoc Interaction Test and Subset Analyses

**Figure 3:**
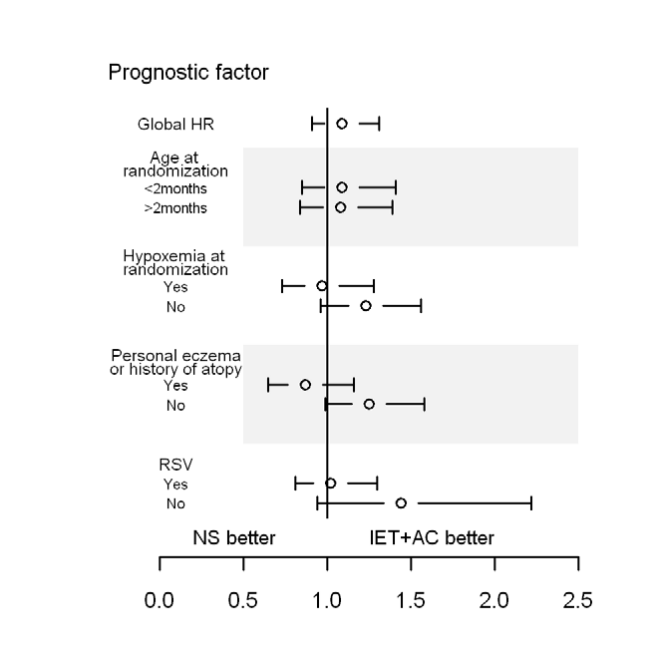
HRs and 95% CIs for healing in the group receiving IET + AC, as compared with the NS group, as a function of baseline prognostic factors. History of atopy was defined as the presence of eczema or asthma in first-degree relatives.

No statistically significant quantitative interaction on time to recovery between treatment and eczema or atopy history was found (*p = *0.06) (Figure 3; Table 6). HR (95% CI) was equal to 1.25 (0.99–1.58) for infants without personal eczema or history of atopy and 0.88 (0.66–1.17) for infants with personal eczema or history of atopy. Similarly, there were no differences in HRs as a function of RSV (HR = 1.01, 0.79–1.28 for infants with and 1.43, 0.94–2.16 for infants without RSV infection, *p = *0.15), or of hypoxemia at randomization (HR = 1.23, 0.96–1.56 for infants without and 0.99, 0.75–1.30 for infants with hypoxemia at randomization, *p = *0.25).

**Table 6:** Search for treatment by covariate interactions on the main outcome measure, time to recovery.

Prognostic Baseline Covariate	NS	IET + AC	HR [95% CI] of Recovery	*p*-Value^a^
	Median Time to Recovery, d [95% CI]			
**Personal eczema or history of atopy** b,c				0.06
** Yes (** ***n*** ** = 197)**	1.96 [1.36–2.73]	2.30 [1.73–3.07]	0.88 [0.66–1.17]	
** No (** ***n = *** **293)**	2.42 [2.04–2.85]	2.02 [1.92–2.33]	1.25 [0.99–1.58]	
**RSV** d				0.15
** Positive (** ***n = *** **289)**	2.34 [1.97–2.99]	2.33 [1.94–2.88]	1.01 [0.79–1.28]	
** Negative (** ***n = *** **97)**	2.33 [1.35–3.32]	1.92 [1.29–2.08]	1.43 [0.94–2.16]	
**Hypoxemia at randomization**				0.25
** Yes (** ***n = *** **216)**	2.73 [2.30–3.32]	2.47 [2.02–3.17]	0.99 [0.75–1.30]	
** No (** ***n = *** **273)**	1.90 [1.36–2.52]	1.96 [1.51–2.08]	1.23 [0.96–1.56]	

a *p*-Value refers to the statistics of Gail and Simon's quantitative interaction test (1df-chi-squared test).

b The data were obtained from the parents.

c History of atopy was defined as eczema or asthma in first-degree relatives.

d RSV was tested in only 386 infants.

## Discussion

This study provides an evaluation of chest physiotherapy with IET + AC in a large population of infants hospitalized for a first episode of bronchiolitis with time to recovery as the primary endpoint. In this seven-center trial, no evidence of any difference in time to recovery between the IET + AC group and the NS group was found, with no interaction with age group. The CIs around estimates of effect excluded a clinically meaningful difference in time to recovery between groups in the whole population.

Otherwise, there was no evidence of any difference in secondary outcomes between children with and without IET + AC except for vomiting and transient respiratory destabilization during the procedure. This significant difference of vomiting and respiratory destabilization frequency was expected and in accordance with our physiotherapists'experiences. However, it is worth noting that these side effects of IET + AC were transient and resolved as soon as the procedure was interrupted. Although not statistically significant, the percentage of bradycardia during a procedure was three times higher in the group EIT + AC. This side effect of IET is rare but known, and it is important to note that bradycardia quickly disappeared in all children.

For parents, predictably, the perception of the arduousness of the procedure was significantly higher for IET + AC as compared to NS. Indeed, IET + AC is a tiring procedure for children even though the recovery is quick once the procedure is over. This perception may explain the absence of difference between both groups for the evaluation of the global comfort.

Although we preplanned an economic analysis, it was not conducted given the negative findings on efficacy outcomes.

Chest physiotherapy is recommended in infants with bronchiolitis by the French national consensus guidelines [17]. The rationale for such a recommendation is that physiotherapy could enhance the clearance of bronchial secretions, thereby decreasing respiratory distress and chest recession and increasing oxygenation. Airflow has not been assessed in infants undergoing IET, but the feasibility of achieving maximal flow (i.e., flow limitation at low lung volumes) has been demonstrated in sleeping healthy infants wearing an inflatable jacket compressing the thorax at the end of normal inspiration [16],[25] or after lung inflation to total pulmonary capacity [14],[15]. When the thorax was compressed from total pulmonary capacity to residual volume, flow limitation at low lung volumes (distal airways) was achieved for lower transpulmonary pressure than flow limitation at high lung volume (large airways) [14],[15], but further increase in transpulmonary pressure could have a negative effect on flow limitation at low lung volumes. These studies conducted in healthy sleeping infants do not mimic IET + AC applied in infants with bronchiolitis. In the present study, pulmonary distension could alter chest wall compliance and therefore transpulmonary pressure transmission. Infants were awake with the possibility to decreased thoraco-pulmonary compliance in response to manipulations, and most of all interindividual variability in IET + AC application could not be avoided despite the optimal training and continuous survey of IET + AC during the study. However, IET + AC is routine practice in France and physiotherapists were substantially experienced and trained. Therefore, the study reflects the effectiveness of physiotherapy in infants with bronchiolitis in real life practice as recommended by guidelines.

Previous studies on chest physiotherapy included smaller numbers of subjects, with physician-assessed clinical scores as the primary outcome and nonrelevant variables as secondary outcomes (durations of oxygen supplementation and hospitalization duration stay) [26]–[28]. Duration of oxygen supplementation was clearly not a relevant outcome because it was required in only 56.5% of the infants studied. The results of any study including only infants requiring oxygen supplementation should therefore not be extrapolated to infants not requiring oxygen.

We tried to determine the time to recovery as accurately as possible. It was therefore necessary to assess the conditions of the infants around the clock, and not only when physicians were present. Nurses recorded respiratory signs, heart rate, and SpO_2_ at least every 8 h, as previously performed [29]. Thus, the 0.3-d (which is almost equivalent to 8 h) difference observed between the two randomized groups in median time to recovery could be related to the study design, which hampered the detection of a smaller difference. However, a smaller gain of healing time would not have any clinical relevance. Otherwise, we have previously shown that the clinical signs used for monitoring (respiratory rates and retraction signs) were recorded similarly by all physicians, nurses, and physiotherapists, with a high level of interobserver reproducibility [20]. We compared IET + AC with NS, because NS provides temporary relief for nasal congestion and is routinely used.

Age, which was thought to be a possible determinant of IET efficacy, did not influence treatment effect. We used a cut-off point of 2 mo to allow stratifying randomization according to previous recommendations that set the age limit for severe bronchiolitis between 6 and 8 wk. Indeed, although such a dichotomization achieved some loss of information, the effectiveness of chest physiotherapy could have been decreased in the youngest owing to their highest risk of severe disease. Airway size increases with age, and we thought that it would be difficult to increase the clearance of bronchial secretions from very small airways by physiotherapy. The lack of documented specific mechanisms of pulmonary change after IET makes it difficult to draw firm conclusions concerning the age-related efficacy of physiotherapy.

Post hoc analyses showed no significant quantitative interaction between IET + AC effect on the main endpoint and personal atopy or history of atopy in first-degree relatives, the type of virus, or the presence of baseline hypoxemia. Although exploratory, our findings for infants without personal atopy or history of atopy in first-degree relatives and for those without hypoxemia were close to statistical significance as shown by the CIs around estimate of effect for these subgroups (respectively [0.99–1.58] and [0.96–1.56]) and deserve further investigations.

Infants included in the present study represent a spectrum of bronchiolitis phenotype including infants prone or not prone to experience repeated wheezing episodes. Reynolds et al. [30] suggested that there might be two different groups of patients: those with obstructive disease resulting entirely from infection, thickening of the bronchiolar walls, and intrabronchial secretions, and those with a predisposition to asthma, who develop obstruction because of both inflammation and bronchospasm. It is still currently impossible to determine which of the infants hospitalized for bronchiolitis will subsequently develop asthma, even though family history of atopy and atopic dermatitis in the infant have been shown to be predictive of asthma [31]. It is possible that future studies will lead to recommendations for bronchiolitis treatment according to the phenotype of the infant, rather than merely being based on current disease diagnosis alone [32]. Our study was not designed to address this issue and atopic status was not found to influence the effect of IET + AC.

The physiopathology of bronchiolitis remains unclear, but the need for oxygen supplementation is considered as a marker of considerable ventilation heterogeneity, due to a large number of units with poor ventilation (obstructed) and good perfusion or overdistension and poor perfusion. In the absence of segmental or lobar lung atelectasia, ventilation heterogeneity is a peripheral phenomenon unlikely to be affected by physiotherapy. Infants requiring oxygen supplementation may not benefit at all from physiotherapy, but the benefits of physiotherapy for nonhypoxemic infants did not reach significance (Figure 3; Table 5). Studying a mixture of patients may provide unclear or misleading results. This perspective raises questions about limiting studies to homogeneous populations, making it impossible to extrapolate the results obtained to patients with a different clinical status. The heterogeneity in concerned patient populations may contribute to the difficulty of definitively evaluating physiotherapy in clinical trials and in making clear recommendations on its benefit in practice.

The “interphysiotherapist” variability is an important issue in our study. Despite having taken great care in optimizing the training, some variability in the way compression was applied may remain and is difficult to evaluate. We deliberately chose to have more than one physiotherapist by center to mimic as closely as possible real-life conditions, and to be able to extrapolate our results, should they have been positive. The problem of practitioner variability for complex interventions is common in nonpharmacological trials. We standardized the cessation of the intervention on the same auscultation-efficacy criteria in order to reduce interphysiotherapist variability.

In summary, this large, randomized, controlled, outcomes assessor-blind and parent-blind trial of three daily chest physiotherapy sessions with the IET technique in infants hospitalized for a first episode of bronchiolitis provided no evidence that this treatment shortened time to recovery. Our results did not support the recommendation that chest physiotherapy be routinely performed in hospitalized infants with acute bronchiolitis. Our conclusions in infants with severe bronchiolitis cannot necessarily be extrapolated to infants with mild or moderate bronchiolitis. Further studies would be required to assess chest physiotherapy in outpatients and for infants without history of atopy.

## Supporting Information

Alternative Language Text S1 French translation of the article by VG.
(0.34 MB PDF)

Text S1 Trial protocol.
(0.30 MB PDF)

Text S2 CONSORT checklist.
(0.11 MB PDF)

## Abbreviations

AC: assisted cough
CI: confidence interval
HR: hazard ratio
IET: increased exhalation technique
IQR: interquartile range
NS: nasal suction
PICU: pediatric intensive care unit
RR: relative risk
RSV: respiratory syncytial virus
SD: standard deviation
