# Supplementary material for: Effectiveness of Chest Physiotherapy in Infants Hospitalized with Acute Bronchiolitis: A Multicenter, Randomized, Controlled Trial
Source: PLoS Med. 2010 Sep 28;7(9):e1000345. doi: 10.1371/journal.pmed.1000345 (PMC2946956; doi:10.1371/journal.pmed.1000345)
Supplement: Alternative Language Text S1 — French translation of the article by VG. (0.34 MB PDF) [file pmed.1000345.s001.pdf]

Efficacité de la kinésithérapie respiratoire chez les nourrissons hospitalisés pour bronchiolite aiguë: etude multicentrique randomisée controlée.

Traduction Française de l'article **Effectiveness of Chest Physiotherapy in Infants Hospitalized with Acute Bronchiolitis: a Multicenter, Randomized, Controlled Trial** publié dans **PLoS Medicine, 2010**

Vincent Gajdos M.D., MPH[1,2,3], Sandrine Katsahian M.D., Ph.D.[4,5], Nicole Beydon M.D.[6], Véronique Abadie M.D., Ph.D.[7,8], Loïc de Pontual M.D., Ph.D.[9,10], Sophie Larrar M.D.[3,11], Ralph Epaud M.D.[12,13], Bertrand Chevallier M.D.[14,15], Sylvain Bailleux[1], Alix Mollet – Boudjemline M.D.[1] Jean Bouyer Ph.D.[2,3], Sylvie Chevret M.D., Ph.D.[4,5], and Philippe Labrune M.D., Ph.D.[1,3]

[1]APHP, Service de Pédiatrie Générale – Hôpital Antoine Béclère – Clamart, [2]Inserm CESP Centre de recherche en Epidémiologie et Santé des Populations, U1018, Equipe Reproduction et développement de l'enfant, Villejuif, [3]Université Paris Sud – 11, [4]APHP, Départment de Biostatistiques et d'Informatique Médicale – Hôpital Saint Louis – Paris, [5]Inserm UMRS U717, [6]APHP, Unité de Pneumologie, Department de Pédiatrie – Hôpital Robert Debré – Paris, [7]Département de Pédiatrie – Hôpital Necker Enfants Malades – Paris, [8]Université Paris 5, [9]APHP, Service de Pédiatrie Générale – Hôpital Jean Verdier – Bondy, [10]Université Paris Nord – Bobigny, [11]APHP, Pediatric Emergency Department – Necker Enfants Malades – Paris, [12]APHP, Service de Pneumologie Pédiatrique – Hôpital Armand Trousseau, Paris, [13]Université Paris 6, [14]APHP, Service de Pédiatrie Générale – Hôpital Ambroise Paré – Boulogne, [15]Université Versailles – Saint Quentin en Yvelines,
Tous en France.

Auteur correspondant: Vincent Gajdos, Hôpital Antoine Béclère, BP 405, 92141 Clamart cedex, France. Tel: +33 1 45 37 42 72. E-mail: vincent.gajdos@abc.aphp.fr

ABSTRACT

Contexte: Le traitement de la bronchiolite aiguë du nourrisson est essentiellement symptomatique, mais la kinésithérapie respiratoire est fréquemment réalisée dans certains pays. En France, une technique spécifique qui combine l'augmentation du flux expiratoire (AFE) et la toux provoquée (TP) a été recommandée. Notre objectif était d'évaluer l'efficacité de la kinésithérapie respiratoire (AFE+TP) chez des nourrissons sans antécédent particulier hospitalisés pour première bronchiolite aiguë.

Méthodes et résultats: Nous avons mené une étude multicentrique (7 centres pédiatriques Français), randomisée, contrôlée dans laquelle les personnes évaluant le critère de jugement et les parents des nourrissons étaient aveugles du traitement reçu par les nourrissons. Nous avons inclus 496 nourrissons hospitalisés pour une première bronchiolite aiguë entre octobre 2004 et janvier 2008. Le traitement reçu par le nourrisson était attribué au hasard et les nourrissons recevaient soit AFE+TP (intervention expérimentale, n=246), soit des aspirations nasales (AN, intervention contrôle, n=250). Ni les parents, ni les personnes évaluant le critère de jugement, ni les investigateurs ne connaissait le type de traitement reçu pas l'enfant (seuls les kinésithérapeutes en avaient connaissance).

Le critère de jugement principal était le délai de guérison défini de la façon suivante : la guérison était déclarée quand était obtenu de manière stable pendant 8 heures une absence d'oxygénodépendance, des signes lutte respiratoire absents ou mineurs et des prises alimentaires supérieurs aux 2/3 des besoins quotidiens.

Les critères de jugement secondaires étaient l'admission en réanimation, la nécessité d'une assistance ventilatoire, d'un traitement antibiotique, la description des effets indésirables survenus pendant la procédure ainsi que l'évaluation par les parents du confort de leur enfant, de la pénibilité de la procédure et de son effet sur l'état respiratoire de leur enfant. L'analyse statistique a été réalisée en intention de traiter.

Le délai médian de guérison était de 2.31 jours, (95% CI, 1.97 à 2.73) pour le groupe contrôle et de 2.02 jours (95% CI, 1.96 à 2.34) pour le groupe recevant l'intervention, ne permettant pas de montrer une efficacité significative de la kinésithérapie respiratoire (HR=1.09, 95% CI, 0.91 à 1.31, p=0.33). Il n'a pas été retrouvé d'interaction entre l'âge et l'intervention sur le délai de guérison (p=0.97). La fréquence des vomissements et des déstabilisations respiratoires transitoires pendant la procédure était plus importante pour le groupe AFE+TP que pour le groupe AN (RR=10.2, 95%CI, 1.3 à 78.8, p=0.005 and RR=5.4, 95%CI, 1.6 à 18.4, p=0.002, respectivement). Il n'y avait pas de différence entre les groupes pour la survenue de bradycardies avec ou sans désaturation pendant la procédure (RR=1.0, 95%CI, 0.2 à 5.0, p=1.00 et RR=3.6, 95%CI, 0.7 à 16.9, p=0.10 respectivement). Les parents considéraient la procédure plus pénible dans le groupe AFE+TP (différence moyenne=0.88, 95%CI, 0.33 à 1.44, p=0.002) alors qu'il n'y avait pas de différence entre les deux groupes en ce qui concerne l'évaluation du confort de leur enfant (différence moyenne= -0.07, 95%CI, -0.53 à 0.38, p=0.40). Il n'y avait pas de différence significative entre les deux groupes en ce qui concerne les admissions en réanimation (RR=0.7, 95%CI, 0.3 à 1.8, p=0.62), la nécessité d'une assistance ventilatoire (RR=2.5, 95%CI, 0.5 à 13.0, p=0.29) ou d'un traitement antibiotique (RR=1.0, 95%CI, 0.7 à 1.3, p=1.00).

Conclusion: Il n'a pas été mis en évidence d'effet de la kinésithérapie respiratoire utilisant la technique de l'augmentation du flux expiratoire associé à la toux provoquée sur le délai de guérison des nourrissons hospitalisés pour une première bronchiolite aiguë. De nouvelles études sont nécessaires pour évaluer l'efficacité de cette technique dans des populations particulières comme les nourrissons traités en ambulatoire et ceux qui n'ont pas d'histoire familiale d'atopie.

(ClinicalTrials.gov numéro NCT00125450)

INTRODUCTION

La bronchiolite aiguë du nourrisson est une maladie virale habituellement spontanément résolutive et qui est le plus souvent liée à une infection par le virus respiratoire syncitial (VRS). Il s'agit de l'infection respiratoire basse la plus fréquente dans les premières années de vie et l'atteinte est le plus souvent légère à modérée. Toutefois, certains nourrissons développent une maladie plus sévère et environ 1% des nourrissons atteints et sans antécédent particulier doivent être hospitalisés [1,2,3]. Des études récentes en population ont montré que l'incidence annuelle des hospitalisations pour une pathologie liée au VRS se situait entre 5 et 17 pour 1000 nourrissons âgés de moins de 12 mois [4,5].

Le traitement de la bronchiolite aiguë du nourrisson est essentiellement symptomatique et comporte selon les situations une oxygénothérapie voire une assistance respiratoire, la réduction au maximum des stimulations et un support nutritionnel entéral voire parentéral.

Les symptômes respiratoires sont la conséquence d'une obstruction bronchiolaire due à un œdème inflammatoire des petites voies aériennes, avec accumulation de mucus et de débris cellulaires secondaires à la nécrose respiratoire causée par le virus [6,7,8].

La kinésithérapie respiratoire est régulièrement utilisée chez les enfants atteints de maladies respiratoires chroniques comme la mucoviscidose ou la dyskinésie ciliaire primitive, ou chez les enfants atteints de pathologies neuromusculaires. L'objectif de cette technique est de faciliter le drainage et l'élimination des secrétions trachéo-bronchiques. Le but ultime de la kinésithérapie est de diminuer l'obstruction des voies aériennes et ce faisant, de diminuer la résistance et ainsi d'améliorer les échanges gazeux, ce qui permet de diminuer le travail respiratoire. Les recommandations internationales ne préconisent pas la kinésithérapie respiratoire dans la prise en charge des nourrissons atteints de bronchiolite aiguë [9,10]. Ces

recommandations sont basées sur une revue récente de la Cochrane Data Base, dont l'analyse de trois essais cliniques [11] ne permet pas de répondre définitivement à la question de l'efficacité de cette technique, et ce pour plusieurs raisons. Une des raisons principales est que ces études évaluaient des techniques de kinésithérapie respiratoire basées sur la percussion et les vibrations. Ces techniques diffèrent énormément de la technique de l'augmentation du flux expiratoire (AFE) associée à la toux provoquée (TP) qui semble plus appropriée dans ce contexte. L'AFE a pour objectif de drainer les voies aériennes distales alors que la TP est reconnue pour faciliter la désobstruction des voies aériennes proximales de plus gros calibre [12,13]. Au cours de l'AFE, la compression manuelle du thorax du nourrisson vise à faciliter le drainage du mucus par la génération d'un débit aérien maximal à bas volume pulmonaire dans les voies aériennes distales (comme lors de la technique d'exploration fonctionnelle respiratoire utilisant la compression thoracique rapide) [14,15,16].

La conférence de consensus Française sur la prise en charge de la bronchiolite aiguë du nourrisson recommande l'utilisation de la kinésithérapie respiratoire utilisant la technique de l'AFE+TP [17]. Bien que la prescription de l'AFE+TP soit très fréquente en France pour tous les nourrissons atteints de bronchiolite aiguë (82.5% à 99%) [18], l'efficacité de cette technique n'a, jusque là, pas été évaluée.

Nous avons donc conduit la première large étude multicentrique, randomisée, contrôlée dans laquelle les personnes évaluant le critère de jugement et les parents des nourrissons étaient aveugles du traitement reçu par le nourrisson, visant à évaluer l'efficacité de la kinésithérapie respiratoire utilisant l'AFE et la TP chez des nourrissons précédemment sains, hospitalisés pour une première bronchiolite aiguë.

METHODES

CONSIDERATIONS ETHIQUES

Le comité de protection des personnes se prêtant à la recherche biomédicale de Saint Germain en Laye a approuvé cette étude.

PATIENTS

Nous avons conduit l'étude dans sept départements de pédiatrie Français localisés à Paris ou dans sa banlieue (A. Béclère – Clamart, J. Verdier – Bondy, A. Paré – Boulogne, K. Bicêtre – Le Kremlin Bicêtre et Paris — R. Debré, Necker – Enfants Malades et A. Trousseau) durant les épidémies de bronchiolite aiguë survenues entre le premier octobre 2004 et le 31 janvier 2008. Les dates de début et de fin d'étude étaient les mêmes pour tous les centres participants.

Nous avons inclus des nourrissons âgés de 15 jours à 24 mois hospitalisés pour un premier épisode de dyspnée sifflante pour lequel le diagnostic de bronchiolite était retenu. Le diagnostic de bronchiolite était posé devant une anamnèse évocatrice d'infection respiratoire haute accompagnée de signes cliniques compatibles avec le diagnostic de bronchiolite (détresse respiratoire avec freinage ou sibilants plus ou moins associés à des râles crépitants).

Les nourrissons étaient éligibles dans les 24 heures qui suivaient leur hospitalisation s'ils présentaient au moins un des critères suivants au moment de l'admission : aspect toxique, survenue d'apnées ou de cyanose, fréquence respiratoire > 60/min, saturation percutanée de l'hémoglobine en oxygène (SpO_2) < 95%, prises alimentaires quotidiennes inférieures à 2/3 des besoins. Le nourrisson pouvait avoir reçu un maximum de 2 séances de kinésithérapie respiratoire entre l'admission et l'inclusion.

N'étaient pas éligibles les nourrissons qui avaient une détresse respiratoire sévère nécessitant une admission immédiate en réanimation, ou une pathologie cardiaque, ou une pathologie pulmonaire chronique ou un antécédent de prématurité (naissance avant 34 semaines d'aménorrhées). De même, les nourrissons qui avaient une contre-indication à la réalisation de l'intervention (AFE+TP) n'étaient pas éligibles : thrombopénie, corticothérapie prolongée, rachitisme, pathologie osseuse, fracture de côte.

Les parents étaient informés des objectifs de l'étude et de son déroulement. Ils étaient en particulier informés du fait que s'ils acceptaient que leur enfant participe, ils ne pourraient pas rester avec leur enfant dans la chambre pendant l'intervention (pour garantir le respect de l'aveugle). La participation d'un nourrisson nécessitait le consentement des deux parents.

Le diagnostic de bronchiolite était confirmé cliniquement à l'inclusion et l'investigateur enregistrait la durée des symptômes avant hospitalisation, puis il évaluait l'état respiratoire, la fréquence respiratoire, la fréquence cardiaque, la température corporelle et la SpO_2 en air ambiant. L'interrogatoire des parents enregistrait de manière standardisée les données relatives à l'histoire médicale et en particulier les antécédents personnels d'eczéma chez le nourrisson et les antécédents d'asthme ou d'eczéma chez les parents et/ou la fratrie ainsi que l'exposition au tabagisme passif au domicile.

RANDOMISATION

La randomisation était réalisée par le kinésithérapeute qui ouvrait une enveloppe scellée numérotée contenant l'allocation au hasard dans l'un des deux bras de traitement. La randomisation avait été générée avant le début de l'étude par le biostatisticien avec le logiciel SAS (SAS Inc., Cary, NC) software. La randomisation était stratifiée sur le centre et dans

chaque centre, sur l'âge (nourrissons âgés de moins de deux mois, nourrissons âgés de deux mois ou plus), en utilisant des blocs de permutation avec des tailles de blocs égale à 4; la taille des blocs n'était pas connue des soignants impliqués dans la prise en charge des nourrissons.

L'ensemble de l'équipe pédiatrique ainsi que les parents n'avaient pas connaissance du bras dans lequel était randomisé l'enfant. L'aveugle a été maintenue jusqu'à la fin du recueil des données. Ainsi, les soignants impliqués dans l'évaluation du critère de jugement principal ou dans la décision de co-intervention n'avaient pas connaissance du traitement reçu par les nourrissons inclus.

INTERVENTION DE L'ETUDE

Le traitement (soit l'intervention à l'étude, soit le contrôle) était réalisé par un kinésithérapeute qui s'isolait dans la chambre avec le nourrisson en occultant les fenêtres avec un store ou un drap pour garantir le respect de l'aveugle. Tous les nourrissons inclus recevaient le traitement trois fois par jour. Dans chaque centre, 4 à 6 kinésithérapeutes spécialement entrainés à la kinésithérapie respiratoire de l'enfant ont participé à l'étude. Les kinésithérapeutes n'étaient pas impliqués dans l'évaluation du délai de guérison.

<u>Groupe des nourrissons recevant l'intervention à l'étude</u> : l'intervention était définie par l'augmentation du flux expiratoire (AFE) associée à la toux provoquée (TP) et si nécessaire, une aspiration nasale douce.

Juste avant de commencer l'étude, un kinésithérapeute sénior a présenté la technique dans chaque centre et tous les kinésithérapeutes participants étaient formellement exercés à la technique. Tout au long de l'étude, un kinésithérapeute référent dans chaque centre s'assurait de l'homogénéité des pratiques et de leur conformité aux recommandations.

L'AFE consistait en la réalisation d'un mouvement synchronisé du thorax et de l'abdomen créé par les mains du kinésithérapeute dès le début de l'expiration. Une main était appliquée sur le thorax et l'autre sur l'abdomen, centrée sur l'ombilic, pour appliquer une contrepression abdominale. Ce geste mettait en compression le thorax et l'abdomen pour créer un flux expiratoire passif.

La manœuvre débutait à la fin du plateau inspiratoire et était poursuivie jusqu'à la fin de l'expiration si la compliance thoraco-pulmonaire du nourrisson le permettait, mais sans aller au-delà de la limite de la résistance du poumon et du gril costal. La compression dynamique du système respiratoire qui en résultait augmentait le débit aérien expiratoire. La procédure était répétée jusqu'à l'obtention d'un critère auditif d'efficacité (diminution ou disparition du wheezing et/ou des ronchi) mais sans dépasser 10 à 15 minutes. La procédure était arrêtée en cas de déstabilisation respiratoire. Si aucune toux spontanée n'était obtenue, celle-ci était déclenchée par la manœuvre de toux provoquée qui consistait en une pression de la trachée au dessus de la fourchette sternale. En cas de nécessité, une aspiration nasale était réalisée en fin de procédure avec une sonde souple pour aspirer les secrétions muqueuses. Tous les nourrissons étaient attentivement surveillés pendant la procédure, notamment grâce à l'enregistrement continu de la SpO_2.

Groupe des nourrissons contrôles : de manière similaire au groupe recevant l'intervention à l'étude, le kinésithérapeute s'isolait dans la chambre avec le nourrisson trois fois par jour. Dans ce groupe, le kinésithérapeute réalisait seulement une aspiration nasale douce (AN) pendant quelques instants pour aspirer les secrétions muqueuses ; il restait ensuite dans la chambre du nourrisson pendant un temps similaire à celui du groupe expérimental sans réaliser d'autres manœuvres supplémentaires sur le nourrisson.

AUTRES TRAITEMENTS

Tous les nourrissons inclus dans cette étude étaient traités de manière homogène pour garantir la qualité des soins et diminuer au maximum la variabilité des résultats imputable à d'autres facteurs que le traitement à l'étude. Des recommandations pour la prescription et l'arrêt de l'oxygénothérapie et du support nutritionnel entéral ou parentéral ont été suivies par tous les centres.

L'oxygénothérapie était indiquée en cas de SpO_2 inférieure à 95% à l'éveil et à 92% pendant le sommeil. Elle était interrompue dès que la SpO_2 remontait de façon stable au dessus de 95% à l'éveil et 92% dans le sommeil. Les infirmières réalisaient des épreuves de sevrage de l'oxygénothérapie au minimum trois fois pas jour pour évaluer la SpO_2 en air ambiant.

Une alimentation orale était administrée autant que possible. Un support nutritionnel entéral par sonde oro-gastrique était proposée aux nourrissons ingérant spontanément moins des 2/3 de leurs besoins quotidiens ou présentant des signes de lutte respiratoire importants, une polypnée (>60/min), une hypoxémie ou une majoration des signes respiratoires pendant l'alimentation. Une hydratation parentérale était indiquée en cas de non amélioration des signes respiratoires malgré la nutrition entérale ou si les prises alimentaires étaient insuffisantes (en particulier en cas de vomissements durant l'alimentation entérale). L'hydratation parentérale était interrompue quand le nourrisson était capable de supporter une alimentation orale. Un médecin et une infirmière évaluaient deux fois par jour la nécessité de recourir à un support nutritionnel entéral ou à une hydratation parentérale.

Les autres traitements comme les bronchodilatateurs, les corticoïdes et les antibiotiques n'étaient pas recommandés, en accord avec les recommandations nationales et internationales [2,3,10,19]. Leur prescription était toutefois autorisée si le clinicien le jugeait nécessaire. Les déviations au protocole ainsi que les prescriptions de traitements concomittants étaient enregistrées.

EVALUATION DES CRITERES DE JUGEMENT

- CRITERE DE JUGEMENT PRINCIPAL

Le critère de jugement principal était le délai entre la randomisation et la guérison. Un enfant était considéré comme guéri si pendant huit heures consécutives, il n'avait pas reçu d'oxygène, que les signes de lutte respiratoire étaient considérés comme mineurs ou étaient absents et que les prises alimentaires étaient supérieures à 2/3 des besoins quotidiens. Les infirmières enregistraient la fréquence cardiaque et la fréquence respiratoire, la SpO_2 et quantifiaient les signes de lutte au moins une fois toutes les huit heures, quand le nourrisson était calme. L'évaluation clinique était basée sur un score clinique pouvant être mesuré de manière reproductible toutes les huit heures, indifféremment par un médecin, une infirmère ou un kinésithérapeute [20].

- CRITERE DE JUGEMENT SECONDAIRE

Premièrement, les kinésithérapeutes recensaient les évènements indésirables survenant durant la procédure : bradycardie (<80/mn) sans désaturation, bradycardie avec désaturation (SpO_2<85%), vomissements, déstabilisation respiratoire transitoire ou accès d'hypotonie nécessitant l'arrêt de la procédure.

Le jour de la sortie de l'hôpital, les parents répondaient à un questionnaire relatif à leur perception du confort de leur enfant et étaient invités à donner leur avis sur l'efficacité de l'intervention sur leur enfant. Comme les parents n'étaient pas autorisés à assister à l'intervention leur avis était global et reposait sur l'observation de leur enfant avant et après le passage du kinésithérapeute et sur leur évaluation globale de l'état de leur enfant durant son séjour hospitalier. Les parents répondaient à plusieurs questions : 1/ durant l'hospitalisation, comment évaluez-vous le confort de votre enfant ? les parents répondaient grâce à une échelle visuelle analogique allant de 0 (très mauvaise) à 10 (excellente), 2/ comment évaluez-vous la pénibilité de l'intervention du kinésithérapeute pour votre enfant ? les parents répondaient grâce à une échelle visuelle analogique allant de 0 (pas pénible du tout) à 10 (très pénible), 3/ pensez-vous que l'intervention du kinésithérapeute a nettement détérioré, plutôt détérioré, pas modifié, plutôt amélioré, ou nettement amélioré le confort de votre enfant et 4/ pensez-vous que l'intervention du kinésithérapeute a nettement détérioré, plutôt détérioré, pas modifié, plutôt amélioré, ou nettement amélioré l'état respiratoire de votre enfant ?

Enfin, les admissions secondaires en réanimation, la nécessité d'une assistance ventilatoire, oul'utilisation d'une antibiothérapie étaient recensées. Les parents étaient contactés par téléphone 30 jours après la sortie de l'hôpital pour recenser les récidives de bronchiolite et le taux de ré-hospitalisations.

ESTIMATION DU NOMBRE DE SUJETS NECESSAIRES

Il n'y avait pas dans la littérature de données disponibles sur le délai moyen de guérison des nourrissons hospitalisés pour bronchiolite aiguë. Pour estimer le nombre de sujets nécessaires, nous avons donc utilisé la durée d'hospitalisation évaluée dans les hôpitaux participant à cette étude sur les années précédentes (moyenne de 6.5 jours et déviation standard de 3.5 jours). Nous avons calculé qu'il fallait inclure 228 enfants (114 dans chaque bras) pour détecter une

diminution du délai de guérison de 20% avec un risque de première espèce de 0.05 et une puissance de 80%. Un des objectifs de cette étude était d'évaluer une différence d'effet de l'intervention selon l'âge c'est-à-dire une possible interaction entre l'intervention et l'âge. Nous avons donc constitué deux groupes de nourrissons, le premier constitué de 228 nourrissons âgés de moins de deux mois et le second de 228 nourrissons âgés de deux mois ou plus (soit 456 au total). Nous avons prévu d'inclure 10% de nourrissons en plus pour s'assurer de disposer de suffisamment d'observations au total, en cas de survenue de sorties d'étude ou de retrait de consentement. Nous avons donc prévu l'inclusion d'un total de 500 nourrissons.

ANALYSE STATISTIQUE

L'analyse a été réalisée en intention de traiter et tous les nourrissons inclus dans l'étude ont été étudiés, y compris les deux nourrissons perdus de vue (un dans chaque groupe).

Les données démographiques ont été exprimées en effectif et pourcentage pour les variables binaires ou les variables qualitatives ordonnées, en moyenne et dérivation standard (SD) pour les variables continues qui suivaient une loi normale et en médiane et interquartile (IQR) pour les variables qui ne suivaient pas une loi normale. Les délais de guérison ont été résumés grâce à la médiane et son intervalle de confiance [21].

Nous avons, dans un premier temps, recherché l'existence d'une interaction avec le groupe d'âge (< 2 mois et ≥ 2 mois) pour le critère de jugement principal en utilisant des modèles de Cox pour chaque groupe d'âge puis en testant l'existence d'une interaction quantitative par le test de Gail et Simon [22]. Aucune interaction avec l'âge n'a été retrouvée (p=0.97), ce qui a permis de réaliser l'analyse sur l'ensemble de la population de l'étude. Ainsi, nous avons estimé les courbes de survie pour le délai de guérison sur l'ensemble de la cohorte en utilisant la méthode de Kaplan Meier et nous les avons ensuite comparées selon le bras de randomisation en utilisant le test du log-rank stratifié sur le groupe d'âge.

Dans un second temps, nous avons réalisé une analyse de survie ajustée sur les variables démographiques pouvant avoir une valeur pronostique (antécédent personnel d'eczéma ou histoire d'atopie chez les parents ou dans la fratrie, âge en mois, hypoxémie au moment de la randomisation, nécessité de recourir à une hydratation parentérale au moment de la randomisation, atélectasie au moment de la randomisation, durée des symptômes, utilisation de mucolytiques avant la randomisation, infection par le VRS) en utilisant un modèle de Cox. L'effet centre, qui correspond à l'existence de différences de niveaux des variables basales et de pratiques médicales selon les centres a été recherché en utilisant des modèles de fragilité [23].

En ce qui concerne les critères de jugement secondaires, nous avons comparé la fréquence de survenue d'évènements indésirables en utilisant le test de Fischer. La nécessité d'admission en réanimation et le cas échéant de ventilation artificielle, la survenue d'atélectasies, de récidive et la nécessité de traitements antibiotiques ou de nouvelle hospitalisation ont été comparés entre les deux groupes avec le test de chi-2 stratifié sur l'âge. Les données des échelles visuelles analogiques ont été comparées avec le test de Wilcoxon.

Enfin, nous avons recherché une interaction entre le critère de jugement principal et les variables pronostiques suivantes : antécédent personnel d'eczéma ou histoire d'atopie chez les parents ou dans la fratrie, hypoxémie au moment de la randomisation ($SpO_2<95\%$), et présence d'une infection par le VRS. Ces analyses n'étaient initialement pas prévues dans le protocole et sont des analyses *post hoc*. Toutes ces interactions quantitatives ont été recherchées grâce au test de Gail et Simon [22].

L'effet du traitement a été mesuré avec le hazard ratio (HR) pour les données de survie, avec le risque relatif (RR) pour les variables binaires et avec la différence moyenne pour les

variables continues. Pour chacune de ces mesures, l'intervalle de confiance à 95% a été calculé. Pour les variables continues qui ne suivaient pas une loi normale, l'intervalle de confiance à 95% a été calculé grâce à la méthode du bootstrap [24]. Tous les tests étaient bilatéraux et les tests étaient considérés comme statistiquement significatifs pour une valeur de p de 0.05 ou moins.

L'analyse statistique a été réalisée avec les logiciels R version 2.10.11 (The R Foundation for Statistical Computing, http://www.R-project.org) et SAS version 9.2 (SAS Inc, Cary, NC).

RESULTATS

PATIENTS

Quatre cent quatre vingt seize nourrissons ont été inclus entre octobre 2004 et janvier 2008 (figure 1) : 246 (49.6%) étaient randomisés dans le bras AFE+TP et 250 (50.4%) dans le bras AN. 153 couples de parents ont refusé que leur enfant participe à l'étude ; les raisons de ce refus étaient soit leur désir de rester avec leur enfant pendant la procédure ce qui entrainait une impossibilité de maintenir l'aveugle, soit le désir que leur enfant reçoive le traitement par AFE+TP.

Au moment de la randomisation, il n'y avait pas de différence notable entre les deux groupes de randomisation pour les variables démographiques, les pourcentages de nourrissons qui étaient hypoxémiques ou qui avaient des difficultés alimentaires, pour le nombre de nourrissons qui avaient une infection par le VRS ou pour la durée des symptômes avant l'hospitalisation (tableau 1). Le pourcentage de nourrissons qui avaient une atélectasie mise en évidence sur la radiographie réalisée à l'admission était plus élevé dans le groupe AN (12.9% versus 7.6%).

Avant la randomisation, 36 (14.4%) nourrissons dans le groupe AN et 47 (19.1%) dans le groupe AFE+TP avaient reçu des inhalations de bronchodilatateurs (Salbutamol). Trente-quatre (13.6%) nourrissons dans le groupe AN et 25 (10.2%) dans le groupe AFE+TP avaient été traités par corticoïdes oraux (béthamétasone).

CRITERE DE JUGEMENT PRINCIPAL

L'intervention de kinésithérapie (AFE+TP) n'avait pas d'effet sur le délai de guérison. Le délai médian de guérison [intervalle de confiance à 95%] était de 2.31 [1.97 – 2.73] jours pour le groupe AN et de 2.02 [1.96 – 2.34] jours pour le groupe AFE+TP (HR [intervalle de confiance à 95%]=1.09 [0.91 – 1.31], p=0.33, figure 2, tableau 2). Une hétérogénéité

significative entre les centres, pour les variables basales et les pratiques médicales, a été observée (p=0.001), ce qui signifie qu'indépendamment de l'intervention évaluée dans cette étude, il existait une différence de délai de guérison entre les centres. Après ajustement sur les variables basales pronostiques, le modèle de Cox à effet aléatoires, qui prend en compte l'effet centre, a permis de montrer que l'effet de l'AFE+TP restait non significatif sur le délai de guérison (HR=1.21 [0.97; 1.49], p=0.09). Il faut noter que l'effet centre persistait, bien que diminué, après prise en compte des variables pronostiques basales (p=0.03).

CRITERES DE JUGEMENT SECONDAIRES

Le tableau 3 résume les évènements indésirables rapportées par les kinésithérapeutes survenus durant les procédures dans les deux groupes : il n'y avait pas de différence significative entre les deux groupes pour le pourcentage de nourrissons qui ont eu un épisode de bradycardie avec désaturation (RR [intervalle de confiance à 95%]=1.0 [0.2 – 5.0], p=1.00) ou sans désaturation (RR=3.6 [0.7 – 16.9], p=0.10). En revanche, le pourcentage de nourrissons qui ont eu soit une déstabilisation respiratoire transitoire, soit un vomissement pendant la procédure était significativement plus important dans le groupe traité par AFE+TP par rapport au groupe traité par AN (RR=10.2 [1.3 – 78.8], p=0.005 and RR=5.4 [1.6 – 18.4], p=0.002 respectivement).

Nous avons obtenu 371 (74.8%) réponses au questionnaire parental (187 dans le groupe AN et 184 dans le groupe AFE+TP). Le tableau 4 résume les réponses des parents quant à leur évaluation de l'influence de l'intervention du kinésithérapeute sur le confort de leur nourrisson et sur son état respiratoire. Selon les parents, la procédure était plus pénible pour leur enfant dans le groupe traité par AFE+TP (différence moyenne [intervalle de confiance à 95%]=0.88 [0.33 – 1.44], p=0.002), alors qu'il n'y avait pas de différence significative concernant l'évaluation du confort de leur enfant entre les deux groupes (différence

moyenne= -0.07 [-0.53 – 0.38], p=0.40). Enfin, il n'y avait pas de différence significative dans l'évaluation par les parents de l'influence de l'intervention sur l'état respiratoire (RR=0.99 [0.90 – 1.08], p=0.89 ou sur le confort de leur enfant (RR=0.99 [0.94 – 1.05], p=0.84).

Dix-sept (3.4%) nourrissons ont été admis en réanimation dont 7(1.4%) ont nécessité une assistance ventilatoire. Il n'y avait pas de différence entre les deux groupes pour le nombre de nourrissons nécessitant une admission en réanimation (RR=0.7 [0.3 1.8], p=0.62) ou une assistance ventilatoire (RR=2.5 [0.5 – 13.0], p=0.29). Soixante-neuf (28.5%) nourrissons dans le groupe AN et 67 (28.6%) dans le groupe AFE+TP ont reçu des antibiotiques (RR=1.0 [0.7 – 1.3], 1.00). 53 nourrissons dans chaque groupe (31.0% dans le groupe AFE+TP) ont eu une récidive dans les 30 jours qui suivaient la sortie d'hospitalisation (RR=1.1 [0.8 – 1.5], p=0.73). Vingt-six nourrissons ont été hospitalisés dans le mois qui suivait la sortie d'hospitalisation (12 dans le groupe AN et 14 dans le groupe AFE+TP; RR=1.2 [0.6 – 2.6], p=0.68) (Tableau 5). Des symptômes respiratoires étaient responsables de l'hospitalisation de 16 nourrissons (6 dans le groupe AN et 10 dans le groupe AFE+TP) sur les 26 cas. La survenue d'une gastro-entérite aiguë était la seconde cause (7/26) de nouvelle hospitalisation. Les trois dernières hospitalisations secondaires étaient consécutives à des affections diverses non respiratoires.

ANALYSE *POST HOC* : RECHERCHE D'INTERACTION ET ANALYSES EN SOUS-GROUPES (TABLEAU 6 ET FIGURE 3)

Il n'a pas été retrouvé d'interaction quantitative statistiquement significative sur le délai de guérison entre le traitement et l'existence d'un eczéma ou d'une histoire familiale d'atopie (p= 0.06). Le Hazard ratio [intervalle de confiance à 95%] était égal à 1.25 [0.99; 1.58] pour

les nourrissons sans eczéma ou histoire familiale d'atopie et 0.88 [0.66; 1.17] pour ceux qui avaient un eczéma ou une histoire familiale d'atopie. De même, il n'y avait pas de différence de hazard ratio en fonction du statut pour le VRS (HR=1.01 [0.79; 1.28] pour les nourrissons avec, et 1.43 [0.94; 2.16] pour les nourrissons sans infection à VRS, p=0.15). Enfin, il n'y avait pas de différence de hazard ratio selon qu'il existait ou non une hypoxémie lors de la randomisation (HR=1.23 [0.96; 1.56] pour les nourrissons sans et 0.99 [0.75; 1.30] pour les nourrissons hypoxémiques lors de la randomisation, p=0.25).

DISCUSSION

Cette étude est à notre connaissance la première à évaluer l'efficacité de la kinésithérapie respiratoire avec AFE+TP dans une large population de nourrissons hospitalisés pour une première bronchiolite et utilisant le délai de guérison comme critère de jugement. Dans cette étude multicentrique (7 centres), nous n'avons pas mis en évidence de différence de délai de guérison entre les nourrissons traités par AFE+TP et ceux traités par AN. Nous n'avons pas non plus mis en évidence d'effet de l'âge sur l'efficacité de l'AFE+TP. L'intervalle de confiance autour de la mesure de l'effet excluait l'existence d'une différence cliniquement significative des délais de guérison entre les deux groupes sur l'ensemble de la population.

Par ailleurs, nous n'avons pas mis en évidence de différence pour les critères de jugement secondaires entre les nourrissons traités ou non par AFE+TP, excepté pour la survenue de vomissements et de déstabilisation respiratoire transitoire pendant l'intervention. Ces deux différences étaient prévisibles au regard de l'expérience des kinésithérapeutes. Toutefois, il est essentiel de noter que les vomissements et la déstabilisation respiratoire étaient toujours des évènements transitoires, spontanément résolutifs dès l'arrêt de l'intervention. Bien que non statistiquement significatif, le pourcentage de bradycardies durant l'intervention était trois fois plus important dans le groupe AFE+TP. Il s'agit là d'un évènement indésirable rare mais connu de l'AFE et il faut noter que les bradycardies ont régressé rapidement chez l'ensemble des nourrissons.

Comme cela était prévisible, la perception par les parents de la pénibilité de l'intervention était significativement plus importante dans le groupe AFE+TP que dans le groupe AN. En effet, l'AFE+TP est une intervention fatigante pour les nourrissons qui récupèrent vite une fois celle-ci terminée. Cela peut expliquer l'absence de différence dans l'évaluation du confort global entre les deux groupes.

Bien que nous ayions initialement prévu la réalisation d'une analyse médico-économique, celle-ci n'a pas été réalisée en raison de l'absence de démonstration d'une efficacité de l'AFE+TP.

La kinésithérapie respiratoire est recommandée par la conférence de consensus française sur la prise en charge de la bronchiolite aiguë du nourrisson [17]. La justification de cette recommandation repose sur le fait que la kinésithérapie respiratoire pourrait augmenter le drainage des secrétions bronchiques et ainsi, diminuer le travail respiratoire et les signes de lutte, tout en améliorant l'oxygénation. L'effet de l'AFE sur le flux aérien n'a pas été évalué chez les nourrissons ayant été traités par AFE mais la possibilité d'atteindre les débits maximaux (i.e. la limitation du flux aérien à faible volume pulmonaire) a été démontrée chez des nourrissons sains endormis dont le thorax était comprimé par un gilet gonflable à la fin d'une inspiration normale [16,25] ou après une inflation pulmonaire jusqu'à la capacité pulmonaire totale [14,15]. Lorsque le thorax était comprimé de la capacité pulmonaire totale à la capacité résiduelle, la limitation du flux aérien à faible volume pulmonaire (voies aériennes distales) était atteinte pour des pressions transpulmonaires plus faibles que celle requise pour la limitation de flux à haut volume pulmonaire (grosses voies aériennes) [14,15], mais une augmentation plus importante de la pression transpulmonaire pourrait avoir un effet délétère sur le débit maximal à faible volume pulmonaire. Ces études conduites chez des nourrissons sains endormis ne reproduisent pas l'AFE+TP effectués chez les nourrissons atteints de bronchiolite aiguë. Dans notre étude, la distension pulmonaire liée à la maladie pouvait altérer la compliance du gril costal et donc la transmission de la pression transpulmonaire. Les nourrissons étaient éveillés et il est possible que la compliance thoraco-pulmonaire ait été diminuée en réaction aux manipulations du kinésithérapeute. Enfin, la variabilité inter-individuelle dans la réalisation de l'AFE+TP ne pouvait pas être totalement réduite, malgré un

entrainement optimal et une surveillance des modalités de la réalisation de l'AFE+TP pendant l'étude. Toutefois, l'AFE+TP est une pratique courante en France, les kinésithérapeutes en ont une grande expérience puisqu'ils réalisent ce geste très régulièrement. Ainsi, cette étude reflète bien l'efficacité de la kinésithérapie respiratoire en pratique clinique courante chez les nourrissons hospitalisés atteints de bronchiolite aiguë.

Les études existantes sur la kinésithérapie respiratoire se singularisent par les petits effectifs de patients inclus et par leur critère de jugement principal qui est est, le plus souvent, un score clinique et non la guérison. Enfin, dans ces études, les critères de jugement secondaires ne sont pas toujours pertinents (durée de l'oxygénothérapie, durée d'hospitalisation) [26,27,28]. En effet, la durée d'oxygénothérapie est un critère qui n'est que partiellement intéressant dans la mesure où l'hypoxémie n'était retrouvée que chez 56.5% des nourrissons inclus. Ainsi, les résultats d'études réalisées uniquement chez des nourrissons oxygéno-dépendants ne peuvent pas être extrapolés aux nourrissons qui ne seraient pas hypoxémiques.

Nous avons essayé de déterminer le délai de guérison aussi précisement que possible. Pour cela, il était indispensable de pouvoir évaluer l'état respiratoire des nourrissons 24 heures sur 24 et non simplement pendant les périodes de présence médicale. Les infirmières ont évalué les signes respiratoires et enregistré la fréquence cardiaque et la SpO_2 au moins toutes les huit heures comme précédemment décrit [29]. Ainsi, la différence médiane de délai de guérison de 0.3 jours (à peu près équivalente à huit heures) observée entre les deux groupes peut en partie être expliquée par le schéma d'étude qui ne permettait pas la détection d'une différence plus petite. Toutefois, une diminution du délai de guérison de moins de huit heures n'aurait pas eu une grande pertinence clinique. Nous avons par ailleurs préalablement montré que les signes cliniques utilisés pour le suivi des nourrissons (fréquence respiratoire et signes de lutte)

étaient évalués de façon similaire par l'ensemble des soignants (médecins, infirmières, kinésithérapeutes) avec une bonne reproductibilité inter-individuelle [20]. Nous avons comparé l'AFE+TP à l'AN car cette dernière permet de lever temporairement l'obstruction nasale et est utilisée en pratique courante.

L'âge qui aurait pu être un déterminant important de l'efficacité de l'AFE n'influencait pas l'efficacité du traitement dans cette étude. Nous avons stratifié les inclusions sur l'âge en choisissant le seuil de 2 mois pour tenir compte de recommandations qui considèrent que la gravité de la maladie est souvent plus importante avant l'âge de 6 à 8 semaines. Ainsi, bien qu'une telle dichotomisation entraîne une perte d'information sur l'effet de l'âge, nous aurions pu observer une efficacité moindre de la kinésithérapie respiratoire chez les nourrissons les plus jeunes en raison du plus grand risque de maladie sévère. En effet, le calibre des voies aériennes augmente avec l'âge et nous faisions l'hypothèse que le petit calibre des voies aériennes des nourrissons les plus jeunes rendrait plus difficile l'augmentation du drainage des secrétions bronchiolaires par la kinésithérapie respiratoire. Compte tenu du peu de documentation sur les mécanismes spécifiques conduisant à la modification de l'état respiratoire après kinésithérapie, il est difficile de tirer des conclusions définitives sur l'influence de l'âge des nourrissons sur l'efficacité de la kinésithérapie.

Les analyses *post hoc* n'ont pas montré d'interaction quantitative significative entre l'effet de l'AFE+TP sur le délai de guérison et la notion d'antécédent personnel d'eczéma ou d'atopie dans la famille (parents, fratrie), le type de virus ou l'hypoxémie à l'inclusion. Bien qu'exploratoires, nos résultats obtenus chez les nourrissons sans antécédent personnel d'eczéma ou d'atopie familiale et ceux chez les nourrissons non hypoxémiques sont proches de la significativité comme le montre l'intervalle de confiance autour de l'estimateur de l'effet

du traitement pour ces sous-groupes (respectivement [0.99 – 1.58] et [0.96 – 1.56]). Cela pourrait inciter à mieux évaluer l'efficacité de la kinésithérapie respiratoire dans ces populations particulières.

Les nourrissons inclus dans la présente étude représentent un spectre phénotypique de bronchiolite qui survient chez des nourrissons dont seulement un certain nombre présenteront ultérieurement des épisodes répétés de dyspnée sifflante.

Il y a des années, Reynolds et al. [30] ont suggéré qu'il pourrait y avoir deux groupes de patients: ceux ayant une maladie obstructive résultant entièrement de l'infection, avec pour conséquence un épaississement des parois des bronchioles et une majoration des sécrétions intrabronchiques, et ceux qui ont une prédisposition à l'asthme, qui développent une obstruction due à l'inflammation et au bronchospasme. Il est, la plupart du temps, impossible de déterminer quel enfant hospitalisé pour une première bronchiolite développera de l'asthme, même si les antécédents familiaux ou personnels d'atopie ont été démontrés être des facteurs prédictifs d'asthme [31]. Il est possible que de futures études aboutissent à des recommandations pour le traitement de la bronchiolite en fonction du phénotype du nourrisson, et non simplement basées sur le seul diagnostic de la maladie [32]. Notre étude n'a pas été conçue pour résoudre ce problème et n'a pas permis de montrer que l'atopie pouvait influencer l'effet de l'AFE+TP.

La physiopathologie de la bronchiolite aiguë du nourrisson n'est pas encore parfaitement claire mais l'oxygénodépendance est considérée comme un marqueur d'importante hétérogénéité de la ventilation due à la présence concomitante de nombreuses alvéoles faiblement ventilées (à cause de l'obstruction) et qui sont bien perfusées alors que d'autres sont bien ventilées (et distendues) et mal perfusées. En l'absence d'atélectasie segmentaire ou lobaire, Il est peu probable que la kinésithérapie puisse jouer un rôle sur l'hétérogénéité

ventilatoire qui est un phénomène très périphérique. Les enfants nécessitant une oxygénothérapie sont donc probablement moins à même de bénéficier de la kinésithérapie ; toutefois, l'effet de la kinésithérapie chez les nourrissons non oxygénodépendants n'atteignait pas la significativité statistique dans notre étude (tableau 5, figure 3). L'étude d'une population hétérogène (atopique, non atopique, hypoxémique, non hypoxémique) peut conduire à des résultats imprécis, voire incorrects pour certaines populations. Cela pose la question des limites des études réalisées sur des populations hétérogènes et plaide pour la réalisation des futures études sur des populations plus homogènes, avec comme corollaires 1) l'impossibilité d'extrapoler les résultats obtenus à des patients différents de ceux inclus dans l'étude, 2) le risque de ne jamais évaluer correctement la kinésithérapie respiratoire du fait du nombre trop élevé d'études à mener.

La variabilité inter-kinésithérapeute est également un point important. Bien qu'ayant pris soin d'optimiser la formation des kinésithérapeutes, une certaine variabilité dans la façon dont la compression thoracique a été réalisée peut persister et est difficile à évaluer. Nous avons délibérément choisi d'impliquer plusieurs kinésithérapeutes par centre afin d'être dans des conditions aussi proches que possible de la vie réelle pour être en mesure d'extrapoler nos résultats, s'ils avaient été positifs. Il s'agit d'un problème général dans les essais non pharmacologiques. Pour réduire au maximum cette variabilité inter-kinésithérapeute, nous avions pris soin de définir clairement les critères auscultatoires permettant de conclure que l'intervention avait été efficace et pouvait être interrompue.

En résumé, cette large étude randomisée, contrôlée, dans laquelle les personnes évaluant le critère de jugement et les parents des nourrissons étaient aveugles pour l'intervention reçue, n'a pas permis de montrer une réduction du délai de guérison des nourrissons hospitalisés

pour première bronchiolite par la réalisation de kinésithérapie respiratoire avec augmentation de flux expiratoire et de toux provoquée à raison de trois séances par jour . Ces résultats ne permettent pas de soutenir la réalisation en pratique courante de kinésithérapie respiratoire chez les nourrissons hospitalisés pour bronchiolite aiguë.

Nos conclusions ne peuvent toutefois pas être extrapolées aux nourrissons qui ont une bronchiolite aiguë peu à modérément sévère. D'autres études sont nécessaires pour évaluer l'effet de la kinésithérapie respiratoire chez les nourrissons traités en ambulatoires et n'ayant pas d'histoire familiale d'atopie.

Nous remercions les **membres du comité indépendant de surveillance**, P. Blanc (Pediatrie Générale, Hôpital de Poissy – Saint Germain en Laye), L. Chevret (Réanimation pédiatrique – Hôpital du Kremlin Bicêtre, Université Paris Sud 11), C. Delacourt (Réanimation pédiatrique – Hôpital Necker–Enfants Malades, Université Paris 5 – René Descartes), P. Hubert (Pediatric Hôpital Necker–Enfants Malades, Université Paris 5 – René Descartes). Nous remercions le **chef de projet**, M. Legrand (APHP – DRRC) et les **assistantes de recherche clinique,** A. Doutres et A. Traoré.

Nous remercions **tous les cliniciens, kinésithérapeutes, infirmières, enfants et leurs parents** qui ont participé à cette étude.

Financement : cette étude a été financée par un programme hospitalier de recherche clinique du ministère Français de la santé (PHRC national AOM 03/123) et par l'Association des Réseaux Bronchiolites (ARB).

Conflit d'intérêt: tous les auteurs ont complete la declaration standardisée de conflits d'intérêt (www.icmje.org/coi_disclosure.pdf, disponible sur demande à l'auteur correspondant) et déclarent (1) n'avoir aucun support financier pour le travail soumis de personne d'autres que leur employeur; (2) n'avoir aucune relation financière avec des entités commerciales qui pourraient avoir eu un intérêt dans le travail soumis dans les 3 dernières années; (3) qu'aucun de leurs conjoint, partenaire ou enfant n'ont de conflit d'intérêt financier en rapport avec cette étude; and (4) n'avoir aucun intérêt financier en rapport avec le travail soumis."

Références

1. Glezen P, Denny FW (1973) Epidemiology of acute lower respiratory disease in children. N Engl J Med 288: 498-505.

2. Bush A, Thomson AH (2007) Acute bronchiolitis. Bmj 335: 1037-1041.

3. Smyth RL, Openshaw PJM (2006) Bronchiolitis. Lancet 368: 312-322.

4. Hall CB, Weinberg GA, Iwane MK, Blumkin AK, Edwards KM, et al. (2009) The burden of respiratory syncytial virus infection in young children. N Engl J Med 360: 588-598.

5. Koehoorn M, Karr CJ, Demers PA, Lencar C, Tamburic L, et al. (2008) Descriptive Epidemiological Features of Bronchiolitis in a Population-Based Cohort. Pediatrics 122: 1196-1203.

6. Wohl ME, Chernick V (1978) State of the art: bronchiolitis. Am Rev Respir Dis 118: 759-781.

7. Aherne W, Bird T, Court SD, Aherne W, Bird TM, et al. (1970) Pathological changes in virus infections of the lower respiratory tract in children. J Clin Pathol 23: 7 - 18.

8. Hall CB (1998) Respiratory syncytial virus. In: Feigin RD, Cherry JD, editors. Textbook of pediatric infectious diseases. Philadelphia: WB Saunders. pp. 2084 - 2111.

9. SIGN (2006) Bronchiolitis in Children : a National Clinical Guideline. Disponible à http://www.sign.ac.uk/pdf/sign91.pdf

10. Subcommittee on Diagnosis and Management of Bronchiolitis (2006) Diagnosis and Management of Bronchiolitis. Pediatrics 118: 1774-1793.

11. Perrotta C, Ortiz Z, Roque M (2007) Chest physiotherapy for acute bronchiolitis in paediatric patients between 0 and 24 months old. Cochrane Database Syst Rev: CD004873.

12. King M, Brock G, Lundell C (1985) Clearance of mucus by simulated cough. J Appl Physiol 58: 1776-1782.

13. Sivasothy P, Brown L, Smith IE, Shneerson JM (2001) Effect of manually assisted cough and mechanical insufflation on cough flow of normal subjects, patients with chronic obstructive pulmonary disease (COPD), and patients with respiratory muscle weakness. Thorax 56: 438-444.

14. Feher A, Castile R, Kisling J, Angelicchio C, Filbrun D, et al. (1996) Flow limitation in normal infants: a new method for forced expiratory maneuvers from raised lung volumes. J Appl Physiol 80: 2019-2025.

15. Hayden MJ, Sly PD, Devadason SG, Gurrin LC, Wildhaber JH, et al. (1997) Influence of driving pressure on raised-volume forced expiration in infants. Am J Respir Crit Care Med 156: 1876-1883.

16. Marsh MJ, Fox GF, Hoskyns EW, Milner AD (1994) The Hering-Breuer deflationary reflex in the newborn infant. Pediatr Pulmonol 18: 163-169.

17. (2001) Consensus conference on the management of infant bronchiolitis. Paris, France, 21 September 2000. Proceedings. Arch Pediatr 8 Suppl 1: 1s-196s.

18. Beauvois E (2001) Rôle de la kinésithérapie respiratoire dans le traitement de la bronchiolite aiguë du nourrisson. Arch Pediatr 8 Suppl 1: 128S-131S.

19. Yanney M, Vyas H (2008) The treatment of bronchiolitis. Arch Dis Child 93: 793-798.

20. Gajdos V, Beydon N, Bommenel L, Pellegrino B, de Pontual L, et al. (2009) Inter-observer agreement between physicians, nurses, and respiratory therapists for respiratory clinical evaluation in bronchiolitis. Pediatric Pulmonology 44: 754-762.

21. Bland M (2000) An introduction to medical statistics. Oxford: Oxford University Press.

22. Gail M, Simon R (1985) Testing for qualitative interactions between treatment effects and patient subsets. Biometrics 41: 361-372.

23. Aalen OO (1988) Heterogeneity in survival analysis. Stat Med 7: 1121-1137.

24. Efron B, Tibshirani R (1998) An introduction to the bootstrap. Boca Raton ; London: Chapman & Hall/CRC. xvi, 436 p.

25. Hoskyns EW, Milner AD, Hopkin IE (1987) Validity of forced expiratory flow volume loops in neonates. Arch Dis Child 62: 895-900.

26. Bohe L, Ferrero ME, Cuestas E, Polliotto L, Genoff M (2004) [Indications of conventional chest physiotherapy in acute bronchiolitis]. Medicina (B Aires) 64: 198-200.

27. Nicholas KJ, Dhouieb MO, Marshall TG, Edmunds AT, Grant MB (1999) An evaluation of chest physiotherapy in the management of acute bronchiolitis. Physiotherapy 85: 669-674.

28. Webb MS, Martin JA, Cartlidge PH, Ng YK, Wright NA (1985) Chest physiotherapy in acute bronchiolitis. Arch Dis Child 60: 1078-1079.

29. Wainwright C, Altamirano L, Cheney M, Cheney J, Barber S, et al. (2003) A multicenter, randomized, double-blind, controlled trial of nebulized epinephrine in infants with acute bronchiolitis. N Engl J Med 349: 27-35.

30. Reynolds EO, Cook CD (1963) The Treatment of Bronchiolitis. J Pediatr 63: 1205-1207.

31. Castro-Rodriguez JA, Holberg CJ, Wright AL, Martinez FD (2000) A clinical index to define risk of asthma in young children with recurrent wheezing. Am J Respir Crit Care Med 162: 1403-1406.

32. Frey U, von Mutius E (2009) The challenge of managing wheezing in infants. N Engl J Med 360: 2130-2133.

Figure 1. Eligibilité, randomisation et suivi des nourrissons ayant participé à l'étude.

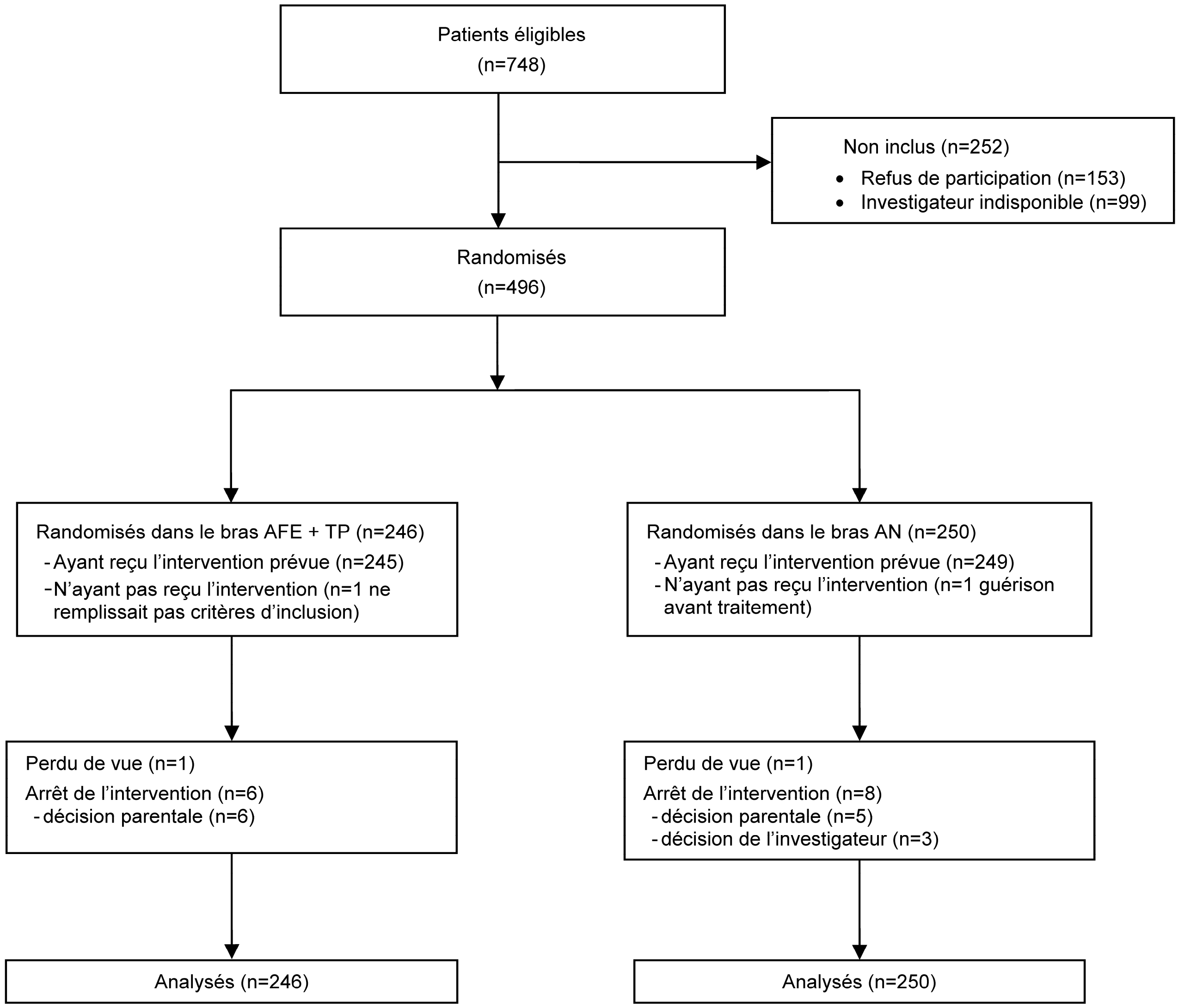

Figure 2. Estimation selon la méthode de Kaplan-Meier de la proportion de nourrissons ne remplissant pas les critères de guérison en function du temps et selon le bras de randomisation

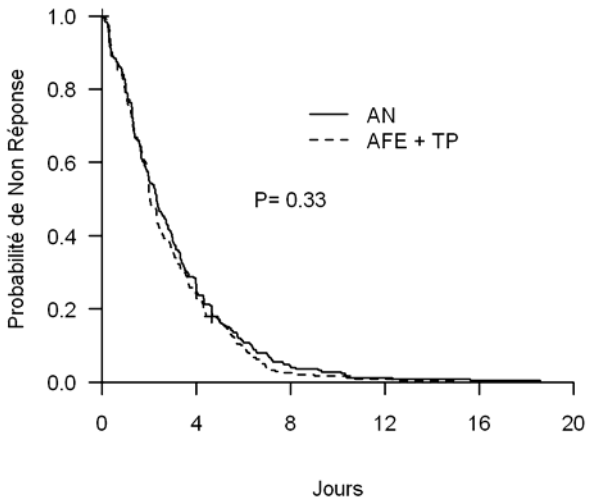

Figure 3. Hazard ratios et leurs intervalles de confiance à 95% des délais de guérison dans le groupe de nourrissons recevant le traitement par AFE+TP compare au groupe AN en function des facteurs prognostic

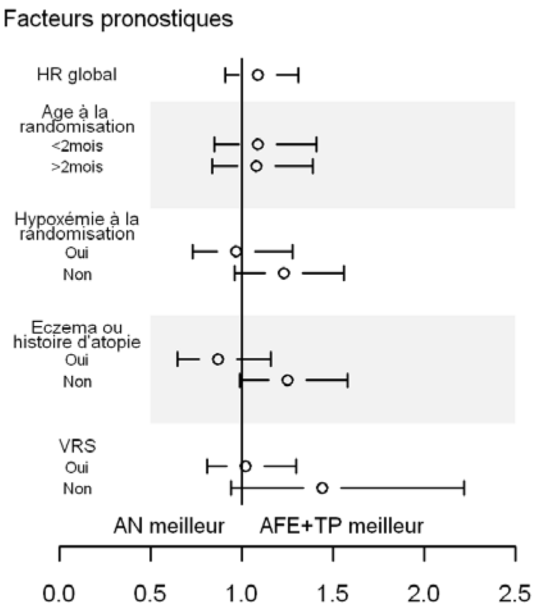

L'histoire d'atopie est définie par un antecedent personnel d'eczéma ou d'eczéma ou d'atopie dans la fratrie.

Tableau 1. Caractéristiques démographiques des nourrissons à leur admission à l'hôpital

	**AN*** **(n=250)**	**AFE+TP*** **(n=246)**
**Age (mois) – médiane [IQR]**	2.0 [1.3 – 4.0]	2.1 [1.3 – 3.8]
**Garçons – n (%)**	141 (56.4)	134 (54.5)
**Terme de naissance (semaine) – moyenne ± SD**	39.1 ±1.65	39.1 ± 1.67
**Tabagisme passif† – n (%)**	69 (29.0)	65 (26.9)
**Eczéma ou histoire familiale d'atopie† – n (%)**	100 (40.7)	97 (39.8)
**Garde en crêche – n (%)**	37 (15.0)	29 (12.0)
**Bronchodilatateurs avant la randomisation – n (%)**	36 (14.4)	47 (19.1)
**Corticosteroides avant la randomisation – n (%)**	34 (13.6)	25 (10.2)
**Difficultés alimentaires avant la randomisation† – n (%)**	222 (89.2)	207 (84.8)
**Durée des symptômes au moment de la randomisation en jours† – médiane [IQR]**	3.0 [2.0 – 4.0]	3.0 [2.0 – 4.0]
**SpO_2<95% au moment de la randomization – n (%)**	110 (44.2)	106 (44.2)
**Atélectasie au moment de la randomisation‖‡ – n (%)**	31 (12.9)	18 (7.6)
**Supplemention en oxygène et hydratation parentérale – n (%)**		
**Pas d'oxygène**	141 (56.4)	135 (54.9)
**Oxygène seulement**	86 (34.4)	86 (34.9)
**Oxygène et hydratation parentérale**	23 (9.2)	25 (10.2)
**VRS positif – n (%)**	152 (76.4)	137 (73.3)
**Température à la randomisation (°C) – moyenne ± SD**	37.2 ±0.7	37.3 ±0.6

¶IQR = Intervalle interquartile

*AFE + TP = augmentation du flux expiratoire + toux provoquée. AN = aspiration nasale

†Les informations ont été obtenues par un interrogatoire des parents

‖Histoire d'atopie définie par la presence d'un eczema chez le nourrisson ou d'eczema ou d'asthme chez les parents ou dans la fratrie.

‡ 480 (96.7%) ont eu une radiographie de thorax à l'admission

Tableau 2. Estimation de l'effet de la technique de l'augmentation de flux expiratoire associée à la toux provoquée sue le délai de guérison (en jours) en fonction de l'âge (analyse univariée)

	**AN***	**AFE+TP***		
	Délai médian de guérison, jours [95CI*]		HR¶ [95CI§] du délai de guérison	p-value‡
**Population totale (n=496)**	2.31 [1.97 – 2.73]	2.02 [1.96 – 2.34]	1.09 [0.91 – 1.31]	0.33
**< 2 mois (n=238)**	2.64 [2.25 – 3.08]	2.47 [1.98 – 3.31]	1.09 [0.84 – 1.41]	0.51
**≥ 2 mois (n=258)**	2.01 [1.65 – 2.44]	2.00 [1.51 – 2.25]	1.09 [0.85 – 1.40]	0.48

*AFE + TP = augmentation du flux expiratoire + toux provoquée. AN = aspiration nasale
¶Hazard Ratio
§95CI: intervalle de confiance à 95%
‡ test du log-rank (stratifié sur le groupe d'âge pour l'analyse sur toute la population)

Tableau 3. Evènements indésirables pendant la procédure rapportés par les kinésithérapeutes

	**AN*** **(n=250)**	**AFE+TP*** **(n=246)**	**Risque Relatif [95 CI[§]]**	*p*¶
**Bradycardie avec désaturation – n (%)**	3 (1.2%)	3 (1.2%)	1.0 [0.2 – 5.0]	1.00
**Bradycardie sans désaturation – n (%)**	2 (0.8%)	7 (2.8%)	3.6 [0.7 – 16.9]	0.10
**Vomissement pendant la procédure**	1 (0.4%)	10 (4.1%)	10.2 [1.3 – 78.8]	0.005
**Destabilisation respiratoire**	3 (1.2%)	16 (6.5%)	5.4 [1.6 – 18.4]	0.002
**Hypotonie**	0 (0.0%)	2 (0.8%)	NA	0.24

[*]AFE + TP = augmentation du flux expiratoire + toux provoquée. AN = aspiration nasale
[§]95CI: intervalle de confiance à 95%
¶Test exact de Fischer

Table 4. Evaluation des parents concenrnant le confort de leur nourrisson et du retentissement de la procedure sur le confort et l'état respiratoire de leur nourrisson**

	AN* (n=187)	AFE+TP* (n=184)	Moyenne de la Différence [95 CI§]	*p*-value
**Evaluation du confort de votre enfant pendant l'hospitalisation – médiane (IQR)**	7.8 [5.7 – 9.0]	7.5 [6.2 – 8.7]	-0.07 [-0.53 – 0.38]	0.40‡
**Evaluation de la pénibilité de la procédure – médiane (IQR)**	4.3 [2.0 – 6.3]	5.0 [3.0 – 7.1]	0.88 [0.33 – 1.44]	0.002‡
			**Risque Relatif [95CI*]**	
**Influence du passage du kinésithérapeute sur le confort de votre enfant – n (%)**			0.99 [0.90 – 1.08]†	0.89¶
Détérioration	5 (2.7%)	12 (6.5%)		
Aucune modification	25 (13.4%)	19 (10.3%)		
Amélioration	157 (83.9%)	153 (83.2%)		
**Influence du passage du kinésithérapeute sur le statut respiratoire de votre enfant – n (%)**			0.99 [0.94 – 1.05]†	0.84¶
Détérioration	4 (2.1%)	1 (0.5%)		
Aucune modification	8 (4.3%)	12 (6.6%)		
Amélioration	175 (93.6%)	170 (92.9%)		

**371 couples de parents (74.8%) ont complété le questionnaire. Les pourcentages sont calculés dur la population de répondants (n= 371)

*AFE + TP = augmentation du flux expiratoire + toux provoquée. AN = aspiration nasale

§95CI: intervalle de confiance à 95%

‡ Test de Wilcoxon

¶Test exact de Fischer

†Le Risque Relatif a été calculé pour l'amélioration versus (Aucune modification + Aggravation)

Table 5. Critères de jugement secondaires‡

	AN* (n=250)	AFE+TP* (n=246)	Risque Relatif [95 CI§]	*p*-value ¶
**Admission en réanimation – n (%)**	10 (4.1%)	7 (2.9%)	0.7 [0.3 – 1.8]	0.62
**Ventilation – n (%)**	2 (0.8%)	5 (2.0%)	2.5 [0.5 – 13.0]	0.29
**Antibiotiques**	69 (28.5%)	67 (28.6%)	1.0 [0.7 – 1.3]	1.0
**Récidive***	53/182 (29.1%)	53/171 (31.0%)	1.1 [0.8 – 1.5]	0.73
**Ré-hospitalisation***	12/182 (6.6%)	14/171 (8.2%)	1.2 [0.6 – 2.6]	0.68

‡Les données ont été obtenues au cours d'un entretien téléphonique avec les parents réalisé 30 jours après la sortie d'hospitalisation. Nous avons obtenu 353 réponses (71.2%) et les pourcentages sont calculés sur la population de répondants (n= 353).

*AFE + TP = augmentation du flux expiratoire + toux provoquée. AN = aspiration nasale

§95CI: intervalle de confiance à 95%

¶ Test exact de Fischer pour la comparaison des pourcentages

Table 6. Recherche d'une interaction entre le traitement et les covariables pronostiques sur le délai de guérison

	**AN***	**AFE+TP***		
	**Délai médian de guérison, days [95CI§]**		**HR [95CI§] de guérison**	***p*-value ‡**
**Eczema ou histoire d'atopie†¶**				0.06
**oui (n = 197)**	1.96 [1.36 – 2.73]	2.30 [1.73 – 3.07]	0.88 [0.66 – 1.17]	
**non (n=293)**	2.42 [2.04 – 2.85]	2.02 [1.92 – 2.33]	1.25 [0.99 – 1.58]	
**VRS‡‡**				0.15
**positif (n= 289)**	2.34 [1.97 – 2.99]	2.33 [1.94 – 2.88]	1.01 [0.79 – 1.28]	
**négatif (n= 97)**	2.33 [1.35 – 3.32]	1.92 [1.29 – 2.08]	1.43 [0.94 – 2.16]	
**Hypoxémie lors de la randomisation**				0.25
**oui (n=216)**	2.73 [2.30 – 3.32]	2.47 [2.02 – 3.17]	0.99 [0.75 – 1.30]	
**non (n=273)**	1.90 [1.36 – 2.52]	1.96 [1.51 – 2.08]	1.23 [0.96 – 1.56]	

*AFE + TP = augmentation du flux expiratoire + toux provoquée. AN = aspiration nasale

§95CI: intervalle de confiance à 95%

†Les informations ont été obtenues par un interrogatoire des parents

¶Histoire d'atopie définie par la presence d'un eczema chez le nourrisson ou d'eczema ou d'atopie dans la fratrie ou chez les parents

‡la p-value fait reference au test de Gail and Simon pour la recherché d'une interaction quantitativet (chi-2 à 1 ddl)

‡‡Le VRS n'a été recherché que chez 386 nourrissons.
