# Supplementary material for: Effectiveness of Chest Physiotherapy in Infants Hospitalized with Acute Bronchiolitis: A Multicenter, Randomized, Controlled Trial
Source: PLoS Med. 2010 Sep 28;7(9):e1000345. doi: 10.1371/journal.pmed.1000345 (PMC2946956; doi:10.1371/journal.pmed.1000345)
Supplement: Text S1 — Trial protocol. (0.30 MB PDF) [file pmed.1000345.s002.pdf]

BRONKINOU

Evaluation du bénéfice et de la tolérance de la Kinésithérapie respiratoire avec augmentation du flux expiratoire dans la prise en charge de la première bronchiolite du nourrisson

Etude randomisée contrôlée multicentrique en double insu

PHRC National AOM 03 123

Version du protocole : *3.8*
Date : *14/05/2004*

Investigateur coordonnateur : Gajdos Vincent

Centres participants :
Ambroise Paré (Boulogne Billancourt)
Antoine Béclère (Clamart)
Jean Verdier (Bondy)
Kremlin Bicêtre (Kremlin Bicêtre)
Necker – Enfants Malades (Paris)
Armand Trousseau (Paris)
Robert Debré (Paris)

Kinésithérapeute référent : Bailleux Sylvain

Analyse Biostatistique :
Chevret Sylvie
Katsahian Sandrine

Promoteur de l'étude :
Assistance Publique Hôpitaux de Paris
Délégation Régionale à la Recherche Clinique
Hôpital saint Louis
1, rue Claude Vellefaux
75010 Paris

Partenaire associé : Association des Réseaux Bronchiolite

TABLE DES MATIÈRES.

1 RÉSUMÉ DU PROTOCOLE 5
1.1 JUSTIFICATION 5
1.2 OBJECTIF PRINCIPAL 5
1.3 OBJECTIFS SECONDAIRES 5
1.4 CRITERE DE JUGEMENT PRINCIPAL 5
1.5 CRITERES DE JUGEMENT SECONDAIRES 5
1.6 TYPE D'ETUDE - PLAN EXPERIMENTAL 6
1.7 JUSTIFICATION DU NOMBRE DE PATIENTS 6
1.8 ACTES REALISES AU COURS DE L'ETUDE 6
1.9 RESULTATS ATTENDUS ET IMPLICATIONS POTENTIELLES 6
2 JUSTIFICATION DE L'ÉTUDE 6
3 OBJECTIFS DE L'ÉTUDE - HYPOTHÈSE PRINCIPALE TESTÉE 7
3.1 OBJECTIF PRINCIPAL 7
3.2 OBJECTIFS SECONDAIRES 7
4 POPULATION ETUDIEE 8
4.1 DESCRIPTION ET NOMBRE DES PATIENTS 8
4.2 SOURCES DE RECRUTEMENT 8
4.3 CRITERES D'INCLUSION 8
4.4 CRITERES DE NON INCLUSION 9
5 TRAITEMENT UTILISÉ POUR L'ÉTUDE 9
6 PLAN EXPÉRIMENTAL - DÉROULEMENT PRATIQUE DE L'ÉTUDE 9
6.1 TYPE D'ETUDE 9
6.2 INCLUSION DANS L'ETUDE 10
6.3 RANDOMISATION 10
6.4 EVALUATION ET SUIVI 10
6.4.1 Pendant l'hospitalisation 10
6.4.2 Après l'hospitalisation 12
6.5 TRAITEMENTS ASSOCIES 13
Traitements Interdits 13
Traitements Autorisés et répertoriés 13
6.6 ASPECTS LIES A LA SECURITE ET LA PROTECTION DES PERSONNES 13
Définition des événements indésirables. 13
Gestion des événements indésirables. 14
6.7 ORGANISATION DE L'ETUDE POUR LES INVESTIGATIONS 15
6.7.1 Randomisation 15

6.7.2 Gestion des données mesurées ou générées ... 15
6.7.3 Coordination ... 15

7 CRITÈRES DE JUGEMENT DE L'ÉTUDE ... 16

7.1 CRITERE DE JUGEMENT PRINCIPAL ... 16
7.2 CRITERES DE JUGEMENT SECONDAIRES ... 16

8 ANALYSE STATISTIQUE ... 16

8.1 JUSTIFICATION DE L'EFFECTIF ... 16
8.2 STRATEGIE DE LA GESTION ET DE L'ANALYSE DES DONNEES ... 17
8.3 TESTS STATISTIQUES UTILISES POUR CHAQUE ANALYSE ET LEUR JUSTIFICATION ... 18
8.4 LIEU D'ANALYSE DES DONNEES ET LOGICIELS UTILISES ... 18
8.5 RESPONSABLE DES ANALYSES DES DONNEES ... 18
8.6 COMITE INDEPENDANT ... 18

9 ASPECTS MÉDICO-LÉGAUX ... 19

9.1 ENGAGEMENTS DE L'INVESTIGATEUR - BONNES PRATIQUES ... 19
9.1.1 Conservation de la documentation et des rapports ... 19
9.1.2 Fin prématurée de l'étude ... 20
9.1.3 Monitoring ... 20
9.2 ASSURANCE ET CONTROLE DE QUALITE ... 20
9.2.1 Promoteur. ... 21
9.2.2 Consentement éclairé des patients ou volontaires. ... 21
9.2.3 CCPPRB ... 21
9.2.4 Lettre d'intention – Officialisation du début de l'étude. ... 21
9.2.5 Assurance ... 21
9.2.6 Aspects pharmaceutiques ... 22
9.3 CAHIERS D'OBSERVATION ... 22
9.4 MODIFICATIONS ET AMENDEMENTS AU PROTOCOLE : ... 22
9.5 ANONYMAT DES PATIENTS OU VOLONTAIRES INCLUS ... 22
9.6 DUREE DE L'ETUDE ... 22
9.7 ARCHIVAGE DES DOCUMENTS ... 22

10 RAPPORT ET PUBLICATION(S) ... 22

11 RÉFÉRENCES BIBLIOGRAPHIQUES : ... 23

12 ANNEXES ... 24

12.1 CRITERES D'HOSPITALISATION. ... 24
12.2 DEROULEMENT D'UNE SEANCE TYPE DE KINESITHERAPIE. ... 25
12.2.1 Les conditions pour réaliser une AFE : ... 25
12.2.2 Désinfection rhino-pharyngée ... 25
12.2.3 Test de précontrainte ... 25

12.2.4 Description de L'augmentation du flux expiratoire (AFE) 26
12.2.5 Technique de Toux provoquée........ 27
12.2.6 Critères d'efficacité de l'AFE 28
12.2.7 Séquence........ 28
12.3 FEUILLE DE RECUEIL. 30
12.4 PROTOCOLE STANDARDISE D'ASPIRATION NASALE 31
12.5 PROTOCOLE STANDARDISE DE DESINFECTION RHINO-PHARYNGEE 32

1 RÉSUMÉ DU PROTOCOLE

1.1 Justification

La bronchiolite du nourrisson est une pathologie fréquente à l'origine de nombreuses hospitalisations, et de prises en charge ambulatoires. Aucune étude n'a validé l'intérêt de la réalisation de kinésithérapie respiratoire dans cette affection, alors que celle-ci est largement réalisée dans les pays francophones.

Il paraît donc important d'évaluer l'efficacité et la tolérance de ce traitement dans la prise en charge des bronchiolites du nourrisson afin de mieux définir sa place dans cette pathologie et ses indications.

1.2 Objectif principal

Montrer une diminution du délai de guérison de 20% (1 journée) chez les nourrissons atteints d'une première bronchiolite et hospitalisés, grâce au traitement par kinésithérapie respiratoire avec augmentation du flux expiratoire (AFE).

1.3 Objectifs secondaires

- Efficacité de la technique de kinésithérapie respiratoire avec augmentation du flux expiratoire (AFE) en terme de désencombrement.
- Tolérance de la séance de kinésithérapie respiratoire avec AFE.
- Montrer que la kinésithérapie respiratoire avec AFE améliore la tolérance de son état et le confort de l'enfant.
- Analyse économique de la réalisation de kinésithérapie respiratoire dans la première bronchiolite aiguë du nourrisson hospitalisé.

1.4 Critère de jugement principal

Temps nécessaire pour obtenir la guérison.

Celle-ci est définie par l'obtention pendant 8 heures de manière stable (à 2 reprises, à 8H d'intervalle) de l'ensemble des critères suivants :

- normoxie (saturation transcutanée en oxygène supérieure ou égale à 95%) sous air
- autonomie alimentaire en dehors d'une gastro-entérite intercurrente
- score de lutte inférieur ou égal a 1
- fréquence respiratoire normale

1.5 Critères de jugement secondaires

- Variation de la saturation en oxygène au décours de la séance de kinésithérapie respiratoire avec AFE.

- Tolérance de la kinésithérapie respiratoire : recensement des malaises et désaturations survenus au cours de séances de kinésithérapie respiratoire.
- Evaluation parentale en fin d'hospitalisation de la tolérance de la pathologie et du confort de leur enfant par un questionnaire standardisé.

1.6 Type d'étude - Plan expérimental

Etude multicentrique randomisée contrôlée en double aveugle avec deux bras parallèles : groupe contrôle (aspiration nasale simple) vs. Expérimental (association kinésithérapie respiratoire avec augmentation de flux expiratoire et aspiration nasale).

1.7 Justification du nombre de patients

Aux risques α=5% et β=20%, en formation bilatérale, l'étude nécessite l'inclusion de 114 malades par groupe pour démontrer une diminution de la durée moyenne de guérison dans le groupe expérimental de 20% à partir d'une durée moyenne dans le bras contrôle de 6,5 jours (écart-type=3,5 jours).
Dans la mesure où une interaction avec l'âge (<, ≥ 2 mois) est possible, on réalisera deux essais indépendants avec au total 456 inclus (228 dans chaque groupe d'âge).
Dans la mesure où il est impossible d'assurer l'absence de sorties de protocole, il est prévu d'inclure 10% de patients supplémentaires afin de s'assurer à la fin du nombre de sujets nécessaires (soit 500 enfants au total).

1.8 Actes réalisés au cours de l'étude

Kinésithérapie avec augmentation du flux expiratoire associée à l'aspiration nasale.

1.9 Résultats attendus et implications potentielles

Raccourcissement du délai de guérison des premières bronchiolites aiguës du nourrisson grâce à 3 séances quotidiennes de kinésithérapie respiratoire avec augmentation du flux expiratoire.
Les implications potentielles sont multiples : augmentation du confort des nourrissons, diminution de l'exposition des nourrissons aux risques d'infection nosocomiale, retentissement en termes de coût lié à la diminution de la durée d'hospitalisation.

2 JUSTIFICATION DE L'ÉTUDE

La bronchiolite du nourrisson est une pathologie fréquente à l'origine de nombreuses hospitalisations, et de prises en charge ambulatoires. Cette affection est reconnue bénigne par tous, et son traitement est variable selon les pays. De nombreux essais thérapeutiques ont évalué l'intérêt dans cette pathologie de l'administration de corticoïdes, d'aérosols de broncho-dilatateurs, avec des résultats mitigés. La kinésithérapie respiratoire qui est largement utilisée en France selon une technique particulière (Augmentation du flux expiratoire : AFE) n'a fait l'objet que de peu d'études, mais est considérée en France comme partie intégrante du traitement. La conférence de consensus sur cette pathologie la

recommande bien que le niveau de preuve de son efficacité soit faible. Cette même conférence de consensus recommande la réalisation d'études pour évaluer l'efficacité d'une telle pratique. Par contre les recommandations anglosaxones ne reconnaissent pas cette thérapeutique comme efficace (1). De fait, les études évaluant l'efficacité de la kinésithérapie respiratoire dans la prise en charge des bronchiolites du nourrisson sont peu nombreuses, et aucune ne conclut à l'intérêt d'un tel traitement. Par ailleurs, aucune étude n'a évalué la technique actuellement proposée en France basée sur l'augmentation du flux expiratoire, qui tient compte des spécificités de la physiologie respiratoire des nourrissons (2).

Aucune étude n'avait jusqu'à ce jour rapporté d'effets indésirables pour cette thérapeutique. Récemment, a été rapportée la survenue de fractures de côtes secondaires à des séances de kinésithérapie chez des enfants atteints de bronchiolite du nourrisson (3). Tous les praticiens ont déjà constaté la survenue de malaises au cours de séances de kinésithérapie respiratoire se traduisant notamment par des désaturations, des changements de teint et des hypotonies. Par ailleurs, le caractère impressionnant pour l'enfant des séances de kinésithérapie respiratoire est admis de tous et le coût d'un tel traitement dans une pathologie fréquente est important et ne peut être négligé.

Il paraît donc important d'évaluer l'efficacité de ce traitement dans la prise en charge des bronchiolites du nourrisson afin de mieux définir sa place dans cette pathologie et ses indications. Cette évaluation a même été recommandée dans une conférence de consensus récente sur la bronchiolite du nourrisson (4, 5).

3 OBJECTIFS DE L'ÉTUDE - HYPOTHÈSE PRINCIPALE TESTÉE

3.1 Objectif principal

Montrer une diminution du délai de guérison de 20% (1 journée) chez les nourrissons hospitalisés pour une première bronchiolite, grâce au traitement par kinésithérapie respiratoire avec augmentation du flux expiratoire.

3.2 Objectifs secondaires

- Montrer l'efficacité de la kinésithérapie respiratoire avec augmentation du flux expiratoire en termes de désencombrement.
- Evaluer la tolérance de la kinésithérapie respiratoire avec augmentation du flux expiratoire, sur la survenue de désaturations et de malaises graves au décours des séances de kinésithérapie respiratoire avec AFE.
- Questionnaire de confort de l'enfant rempli par les parents de l'enfant.
- Analyse économique de la réalisation de kinésithérapie respiratoire dans la première bronchiolite aiguë du nourrisson hospitalisé.

4 Population étudiée

4.1 Description et nombre des patients

Deux populations seront considérées, l'une composée des enfants de moins de deux mois et l'autre de ceux de deux mois et plus.
Cette stratification est justifiée par le fait que l'ensemble des experts consultés s'accordent sur le fait que le bénéfice de cette intervention n'est pas le même dans ces deux populations, c'est à dire qu'il existe potentiellement une interaction entre l'effet de la kinésithérapie et l'âge de l'enfant.
Dans chacune de ces deux tranches d'âge, il est prévu d'inclure 114 enfants par groupe (voir section « Justification du nombre d'inclus »). Il est prévu 10% d'inclusions supplémentaires pour palier aux éventuelles sorties de protocole (125 enfants par groupe et par tranche d'âge).

4.2 Sources de recrutement

Enfants éligibles se présentant aux urgences pédiatriques des Hôpitaux Antoine Béclère (AP–HP, Clamart), Kremlin Bicêtre (AP–HP, le Kremlin Bicêtre), Robert Debré (AP–HP, Paris), Ambroise Paré (AP-HP, Boulogne Billancourt), Jean Verdier (AP-HP, Bondy), Necker (AP-HP, Paris) et Trousseau (AP-HP, Paris) présentant une première bronchiolite du nourrisson et justifiant d'une hospitalisation selon les critères d'hospitalisation définis dans la conférence de consensus sur la bronchiolite du nourrisson (Prise en charge de la bronchiolite du nourrisson. Conférence de consensus - 21 septembre 2000), après consentement éclairé des parents.

4.3 Critères d'inclusion

Sera considéré comme éligible tout enfant présentant les trois critères suivants :

- Nourrisson de plus de 15 jours et de moins de 24 mois
- Avec premier épisode de dyspnée sifflante (wheezing) ou freinage expiratoire
- Ayant une indication au moins d'hospitalisation selon la conférence de consensus (Annexe 1) et présentant au moins une des caractéristiques suivantes :
    - Aspect toxique
    - Apnées – cyanose
    - Tachypnée (>60/min) sous air
    - Saturation transcutanée en oxygène < 95% sous air
    - Troubles digestifs (prises < 2/3 des besoins)
- Inclusion « secondaire » : si un enfant ne remplit pas les critères de gravité sus-cités (aspect toxique, apnées – cyanoses, tachypnée sous air, désaturation sous air, trouble digestif) et que l'un d'eux apparaît dans les 48 premières heures de l'hospitalisation, l'enfant est alors incluable s'il n'a pas reçu plus de deux séances de kinésithérapie respiratoire depuis le début

de son hospitalisation. L'inclusion doit alors être faite sans délai, dans les huit heures qui suivent l'apparition de ces signes.

4.4 Critères de non inclusion

Ne seront pas inclus les enfants présentant au moins l'un des critères suivants :

- Age inférieur à 2 semaines
- Antécédents de prématurité (34 semaines d'aménorrhée ou moins), de dysplasie broncho-pulmonaire
- Cardiopathie sous-jacente, pathologie pulmonaire chronique grave
- Antécédents de bronchiolite
- Indication à un transfert en réanimation
- Réalisation de trois séances de kinésithérapie entre la décision d'hospitalisation et l'inclusion
- Refus de consentement par les parents
- Contre indication à la réalisation de Kinésithérapie respiratoire :
  - Corticothérapie prolongée (plus de un mois)
  - Rachitisme
  - Ostéogenèse imparfaite
  - Thrombopénie
  - Fracture de côte

5 TRAITEMENT UTILISÉ POUR L'ÉTUDE

Le traitement évalué est la séance de kinésithérapie respiratoire avec AFE telle que définie par la conférence de consensus de Lyon qui sera réalisée de manière standardisée (Annexe 2).

6 PLAN EXPÉRIMENTAL - DÉROULEMENT PRATIQUE DE L'ÉTUDE

6.1 Type d'étude

Etude randomisée multicentrique en double aveugle, évaluant l'association kinésithérapie respiratoire avec augmentation de flux expiratoire et aspiration nasale versus aspiration nasale simple, en deux groupes parallèles, avec bénéfice individuel direct. Le double aveugle concerne les parents et les soignants (en sont donc exclus l'enfant lui-même et les kinésithérapeutes).

6.2 Inclusion dans l'étude

L'enfant sera inclus dès son admission dans le service participant sur la période d'étude après vérification des critères d'inclusion et de non inclusion, et notamment après recueil du consentement éclairé signé des parents par l'investigateur.

Une radiographie de thorax sera réalisée à l'inclusion.

Un enfant ne pourra être inclus que si le délai entre l'hospitalisation et la randomisation n'excède pas 24 heures. Ce délai est porté à 48 heures pour les inclusions « secondaires ».

Schéma temporel d'inclusion et de randomisation

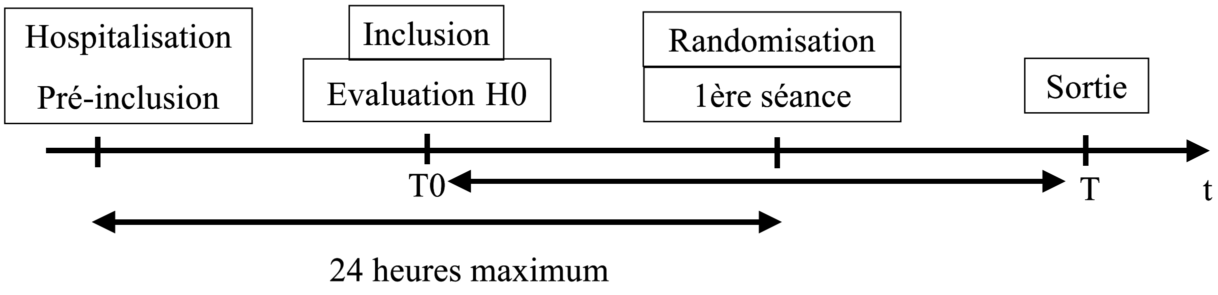

6.3 Randomisation

La randomisation sera réalisée par le kinésithérapeute après inclusion de l'enfant. Elle sera effectuée par l'intermédiaire d'enveloppes cachetées opaques. Seuls les kinésithérapeutes auront connaissance du traitement reçu par l'enfant.

La randomisation sera stratifiée par centre et par groupe d'âge (< 2 mois, ≥ 2 mois).

Pour le groupe expérimental : l'enfant recevra trois séances de kinésithérapie respiratoire avec AFE suivies d'une aspiration nasale (Annexe 4). Ce type de séance sera standardisée, et réalisée de manière identique dans tous les centres.

Pour le groupe contrôle : le kinésithérapeute passera le même temps avec l'enfant, et se contentera de réaliser l'aspiration nasale.

6.4 Evaluation et suivi

6.4.1 Pendant l'hospitalisation

Chaque enfant sera évalué trois fois par jour par son infirmière après qu'il a passé cinq minutes au calme en air ambiant si son état le permet ; si pendant la période de sevrage avant l'évaluation, la saturation transcutanée en oxygène descend en dessous de 90%, l'évaluation sera immédiatement réalisée et l'enfant sera remis sous oxygène. Seront recueillis la saturation transcutanée en oxygène

sous air, la qualité de l'alimentation depuis la dernière évaluation, la fréquence respiratoire et la fréquence cardiaque, le score de lutte et la présence ou non d'une sibilance audible à l'oreille. Cette évaluation sera consignée sur une grille standardisée (Annexe 3).

Cette évaluation aura lieu au moins deux heures après la dernière intervention, et à au moins une heure après le dernier repas.

Les évaluations s'arrêtent quand l'enfant remplit les critères de sortie d'étude. A ce moment, un questionnaire évaluant la tolérance respiratoire et le confort de l'enfant pendant la durée d'inclusion est rempli par l'un des parents.

Les modalités d'alimentation seront standardisées de la façon suivante :

L'enfant sera alimenté au mieux après la séance de kinésithérapie respiratoire. Un délai de 1 heure 30 devra être respecté entre l'alimentation et la séance de kinésithérapie suivante.

<u>Les besoins nutritionnels</u> sont définis ainsi :

- ≤ 9 mois : 100-120 ml/kg
- > 9 mois : 90-100 ml/kg

Le mode d'alimentation sera choisi initialement par l'investigateur. L'enfant recevra dans la mesure du possible une alimentation orale. Dans certaines situations décrites ci-dessous, un gavage gastrique de 1h ou 2h sera prescrit. Si celui-ci est mal toléré, une perfusion intraveineuse le remplacera (QS infra). Dans tous les cas, la nécessité d'un gavage gastrique ou d'une alimentation parentérale sera réévaluée toutes les 12 heures.

<u>Indication d'un gavage</u> :

- prises alimentaires orales inférieures à 2/3 des besoins quotidiens.
- Signes de lutte importants ou majoration de ceux-ci lors de l'alimentation.
- Mauvaise tolérance de l'alimentation : apparition de signes de lutte importants, tachypnée (>60/min), désaturations (saturation <90% sous oxygène), malaises.

<u>Indications d'un arrêt alimentaire et de la pose d'une perfusion</u> :

- Echec du gavage alimentaire :
    - rejets de sonde dans une situation d'alimentation orale impossible.
    - Vomissements (lors d'au moins deux gavages consécutifs)
    - Mauvaise tolérance du gavage : apparition de signes de lutte importants ou majoration de ceux-ci lors du gavage, tachypnée (>60/min), désaturations (< 90% sous oxygène), malaises.

- Situation clinique dans laquelle l'état de l'enfant paraît d'emblée particulièrement grave et où toute alimentation paraît augmenter le risque d'un passage en réanimation (épuisement, trouble de conscience, tachypnée majeure et/ou besoins en oxygène. La reprise d'une alimentation par gavage devra être régulièrement envisagée en fonction de l'amélioration clinique de l'enfant.

Désescalade : la réintroduction d'un gavage et/ou d'une alimentation orale simple sera reconsidérée toutes les 12 heures : elle sera tentée dés que les indications du gavage ou de la perfusion auront disparues. L'investigateur local sera libre de proposer cette désescalade dans des délais plus courts si cela lui paraît cliniquement pertinent.

Les modalités d'oxygénothérapie : Des épreuves de sevrages pluriquotidiennes paraissent indispensables. Des recommandations seront rédigées comme suit :

Indications d'une oxygénothérapie :

- majoration de la détresse respiratoire lors de l'alimentation.
- Saturation en oxygène sous air strictement inférieure à 95% de manière durable (94% ou moins) en phase d'éveil, inférieure à 92% (91% ou moins) en phase de sommeil ou pendant l'alimentation.
- Troubles de conscience

Posologie de l'oxygène : quantité suffisante pour obtenir une saturation supérieure ou égale à 95% en phase d'éveil, supérieure ou égale à 92% en phase de sommeil ou pendant l'alimentation.

Indications du sevrage en oxygène :

- disparition des critères indiquant cette thérapeutique.
- Un essai de diminution du débit ou de sevrage sera réalisé toutes les huit heures. L'oxygène sera maintenu au même débit en cas d'échec de cette épreuve de sevrage.

En cas de passage en réanimation

Il n'est pas prévu de levée d'aveugle. Les réanimateurs sont libres de leurs traitements. Au décours du séjour en réanimation, le suivi de l'enfant reprendra selon les termes du protocole dans son bras de traitement et il sera suivi jusqu'au recueil du critère de jugement principal.

Levée d'aveugle

- En cas de malaise grave (voir p 17)

6.4.2 Après l'hospitalisation

La famille de chaque enfant sera contactée par téléphone par l'attaché de recherche clinique 30 jours après la sortie de l'enfant. Un questionnaire téléphonique recueillera les principaux événements survenus depuis la sortie de l'enfant ;

Les parents recevront une information écrite stipulant qu'en cas de survenue d'un évènement médical dans les 30 jours qui suivent l'hospitalisation, le médecin consulté devra faire une déclaration à l'investigateur avec un questionnaire pré-établi et remis aux parents au moment de la sortie de l'enfant.

6.5 Traitements associés

Traitements Interdits

Aucun traitement n'est interdit.

Traitements Autorisés et répertoriés

- Broncho-dilatateurs par voie inhalée
- Corticoïdes par voie générale ou inhalée
- Aspiration nasale (protocole standardisé : Annexe 4)
- Désinfection rhino-pharyngée (protocole standardisé : Annexe 5)

Ces différents traitements feront l'objet d'un recueil d'administration.

6.6 Aspects liés à la sécurité et la protection des personnes

Définition des événements indésirables.

Événements indésirables graves

- Malaise grave défini par une bradycardie inférieure à 80/min, et une saturation transcutanée en oxygène inférieure à 85%
- Décès
- Tout autre évènement qui remplisse l'une des conditions suivantes :
  - Evènement engendrant un handicap important ou permanent
  - Evènement nécessitant une intervention médicale ou chirurgicale pour prévenir des séquelles importantes ou permanentes

Autres événements indésirables

- Malaise non grave : Accès de pâleur associé à une bradycardie inférieure à 80/minutes avec une saturation transcutanée en oxygène sous air supérieure à 85% ou désaturation en dessous de 85% sans bradycardie (ie fréquence cardiaque supérieure ou égale à 80/min)
- Fausses routes
- Vomissements
- Transfert en réanimation

Gestion des événements indésirables.

Tous les événements indésirables devront être consignés dans le cahier d'observation ou le cahier de surveillance de kinésithérapie respiratoire.

La survenue d'un événement indésirable grave fera l'objet d'un rapport sur un formulaire « Fiche de déclaration d'événement indésirable grave » en accord avec les Bonnes Pratiques Cliniques. L'investigateur devra informer dans les 24 heures (48 heures le week-end) le chef de projet puis remplir la fiche de déclaration et la télécopier au promoteur :

- sous 48 heures en cas de décès qui donne lieu de la part du promoteur à une déclaration sous 7 jours calendaires après réception à la Direction Générale de la Santé,
- sous 8 jours dans les autres cas qui doivent être déclarés à la Direction Générale de la Santé sous 15 jours calendaires après réception.

L'investigateur devra pour chaque événement répertorié, émettre sur la Fiche d'événement indésirable grave un avis médical sur la relation pouvant exister entre l'apparition de l'effet indésirable grave et le protocole.

Les bilans cliniques, les examens diagnostiques et les examens de laboratoire appropriés seront mis en route afin d'identifier l'origine de l'événement et les résultats de ces explorations ainsi que l'évolution du trouble seront rapportés.

L'investigateur coordonnateur a la responsabilité d'informer le Comité Consultatif de Protection des Personnes se prêtant à la Recherche Biomédicale de tous les événements indésirables grave survenant pendant l'étude.

Le promoteur a la responsabilité d'informer la Direction Générale de la Santé de tous les événements indésirables graves liés à la recherche.

En cas de malaise pendant l'intervention du kinésithérapeute, l'événement sera noté sur le cahier de surveillance de kinésithérapie de l'enfant. Il sera procédé par le kinésithérapeute comme le veut l'usage clinique, à une information du médecin investigateur en charge de l'enfant sans levée d'aveugle.

Les fausses routes et vomissements seront consignés dans le cahier d'observation de l'enfant.

Arrêt des traitements : après arrêt du traitement quelle qu'en soit la cause (souhait des parents du malade, décision de l'investigateur), les malades seront suivis jusqu'à la fin de l'étude et les critères d'évaluation recueillis.

6.7 Organisation de l'étude pour les investigations

6.7.1 Randomisation

Lorsqu'un investigateur souhaite effectuer la randomisation d'un patient, il remplit la fiche d'éligibilité figurant dans le cahier d'observation dès qu'il dispose de tous les résultats nécessaires à l'inclusion du patient dans l'essai.

La randomisation sera réalisée par le kinésithérapeute présent après réception de la fiche d'éligibilité de l'enfant. La randomisation sera effectuée par l'intermédiaire d'enveloppes cachetées opaques, à partir de listes de randomisation préétablies équilibrées (taille de bloc non mentionnée aux investigateurs et kinésithérapeutes). Seuls les kinésithérapeutes auront connaissance du traitement reçu par l'enfant.

La randomisation sera stratifiée par centre et par groupe d'âge (< 2 mois, ≥ 2 mois).

6.7.2 Gestion des données mesurées ou générées

Les données seront retranscrites dans un cahier d'observation standardisé. Les données devront être rédigées avec un stylo-bille noir (ou bleu), afin de s'assurer de la lisibilité des copies. Les corrections seront faites de telle sorte que les données originales soient lisibles. Ces corrections seront datées et signées (initiales) par la personne habilitée.

A chaque enregistrement de données, le cahier d'observation sera signé et daté par l'investigateur ou le co-investigateur habilité.

Chaque cahier sera adressé au département de biostatistique et informatique médicale (DBIM) de l'hôpital saint Louis, après monitorage sur site par un ARC.

6.7.3 Coordination

Le suivi du protocole est réalisé par un comité de pilotage coordonné par le Dr Vincent Gajdos et le Pr. Philippe Labrune. Ce comité réunit outre ses coordonnateurs, le promoteur (représenté par le chef de projet en charge de l'étude à la DRRC), le responsable de l'analyse biostatistique et le kinésithérapeute coordonnateur.

Ce comité définira l'organisation générale et le déroulement de l'essai et coordonnera les informations.

Il déterminera initialement la méthodologie et décidera en cours d'essai des conduites à tenir dans les cas imprévus, surveillera le déroulement de l'essai en particulier sur le plan de la tolérance et des évènements indésirables.

7 CRITÈRES DE JUGEMENT DE L'ÉTUDE

7.1 Critère de jugement principal

Temps nécessaire pour obtenir la guérison. Celle-ci est définie par l'obtention pendant 8 heures consécutives (à 2 reprises, à 8H d'intervalle) de l'ensemble des critères suivants :

- normoxie (saturation transcutanée en oxygène supérieure ou égale à 95% à l'éveil) sous air
- autonomie alimentaire en dehors d'une gastro-entérite intercurrente
- score de lutte inférieur ou égal a 1 (annexe 3)
- fréquence respiratoire normale

7.2 Critères de jugement secondaires

- Variation de la saturation transcutanée en oxygène, de la fréquence respiratoire, des signes de lutte et de la sibilance avant/après réalisation de la kinésithérapie respiratoire avec AFE : trois mesures effectuées par les kinésithérapeutes : juste avant la séance, juste après et une heure après
- Survenue de malaises (graves ou non) au décours des séances de kinésithérapie respiratoire avec AFE.
- Evaluation parentale en fin d'hospitalisation de la tolérance de la pathologie et du confort de leur enfant par un questionnaire standardisé (QUALIN modifié).

8 ANALYSE STATISTIQUE

8.1 Justification de l'effectif

Le calcul du nombre de patients nécessaire a été réalisé à partir des données des années précédentes en terme de durée moyenne d'hospitalisation (aucune donnée n'étant disponible sur la mesure du critère principal, par ailleurs lié à la décision de sortie d'hôpital). Compte tenu de la population incluse sur les hôpitaux participants (Antoine Béclère, Robert Debré et Kremlin Bicêtre, Ambroise Paré, Poissy-saint Germain, Louise Michel, Jean Verdier), on peut estimer que le nombre moyen de jours d'hospitalisation est de 6,5 jours (SD =3,5 jours) dans le groupe contrôle.

Aux risques α=5% et β=20%, en formation bilatérale, et selon une procédure d'analyse unique, l'étude nécessite l'inclusion de 114 malades par groupe pour démontrer une diminution de la durée moyenne de guérison dans le groupe expérimental de 1,3 jours (20%) (même écart-type).

Dans la mesure où l'essai s'intéresse à la recherche d'une potentielle interaction avec l'âge, deux groupes de 228 enfants (l'un de moins de 2 mois, l'autre de 2 mois et plus) seront constitués, soit au total 456 enfants inclus.

Dans la mesure où il est impossible d'assurer l'absence de sorties de protocole, il est prévu d'inclure 10% de patients supplémentaires afin de s'assurer à la fin du nombre de sujets nécessaires (soit 500 enfants au total).
Dans la mesure où une analyse intermédiaire pourrait conclure à la nécessité d'une augmentation du nombre de sujets à inclure, il est prévu une adaptation du nombre de sujets en cours d'étude après analyse intermédiaire des données.

8.2 Stratégie de la gestion et de l'analyse des données

Le responsable de la gestion et analyse des données est le département de biostatistique et informatique médicale de l'hôpital saint Louis (Paris).
Après monitoring, le traitement des données sera réalisé conformément aux principes directeurs annexes des systèmes informatiques du Guide des Bonnes Pratiques Cliniques de la Communauté Européenne. Plus précisément, les données seront saisies selon une procédure de double saisie, sous un gestionnaire de bases de données type ACCESS pour Windows. Elles seront validées après saisie, avant le début de l'analyse statistique (à partir de l'émission de requêtes auprès des ARC et cliniciens impliqués dans l'étude).

L'analyse statistique sera réalisée selon le principe de l'intention de traitement : le traitement pris en compte dans l'analyse sera celui défini par tirage au sort, même si ce traitement n'a pas pu être mis en œuvre, quelle qu'en soit la raison. Elle reposera sur le principe de la date de point : tous les enfants inclus avant une date donnée (calendaire) seront analysés (en intention de traiter).

Les préoccupations éthiques justifient la réalisation d'analyses intermédiaires avant l'obtention du nombre d'inclus terminal. Afin de se prémunir d'erreurs d'interprétations souvent observées dans la pratique d'analyses intermédiaires, ces analyses répétées utiliseront la procédure décrite par Pocock (1986). Ces analyses ne porteront que sur le critère de jugement principal d'efficacité et le critère de tolérance. Au terme de chaque analyse intermédiaire non conclusive, les résultats ne seront pas communiqués pour respecter la poursuite de l'aveugle de l'essai. Les résultats de ces analyses intermédiaires seront présentés au comité indépendant de l'essai (voir plus loin).
Une analyse intermédiaire sera réalisée toutes les 250 inclusions (1 analyse intermédiaire).

L'analyse s'intéressera à la question de l'efficacité et de la tolérance de la kinésithérapie respiratoire, globalement, puis recherchera une interaction avec le groupe d'âge.

8.3 Tests statistiques utilisés pour chaque analyse et leur justification

- En absence de censure (c'est à dire dans la mesure où l'on dispose à la date d'analyse d'un délai de guérison pour tous les inclus), on utilisera des tests de rang non paramétriques pour les données continues, et un test exact de Fisher pour les données discrètes ; des modèles de régression adaptés permettront d'ajuster la comparaison thérapeutique sur les facteurs pronostiques et/ou déséquilibrés entre bras de randomisation.
- En présence de censure (ie, si l'on ne dispose pas du délai de guérison pour au moins un inclus), on précisera les causes de censure (notamment, enfant toujours hospitalisé à la date de point, ou décès) et des méthodes statistiques adaptées seront utilisées : estimateur de Kaplan Meier, test du logrank, et modèle de Cox. Ce dernier permettra d'ajuster la comparaison thérapeutique en tenant compte des facteurs pronostiques.
- Dans tous les cas, un test d'interaction (qualitative et quantitative) de Gail et Simon sera appliqué sur le critère de jugement principal entre les groupes d'âge (< 2 mois, ≥ 2 mois). La mise en évidence d'une interaction qualitative significative pourra conduire à interrompre les inclusions dans le groupe d'âge pour lequel le traitement contrôle (ou expérimental) est délétère.

8.4 Lieu d'analyse des données et logiciels utilisés

L'analyse statistique, tant séquentielle que terminale sera réalisée sur les logiciels SAS (SAS Inc, Cary, NC) et Splus 2000 (MathSoft, Inc, Seattle, WA, USA).

8.5 Responsable des analyses des données

Les responsables de l'analyse statistique sont le Pr Sylvie Chevret et le Dr Sandrine Katsahian (Département de Biostatistique et Informatique Médicale, Hôpital Saint Louis, Paris).

8.6 Comité indépendant

Un comité indépendant composé d'experts en pneumologie pédiatrique et réanimation pédiatrique a validé le protocole et en suivra la réalisation en examinant les études intermédiaires.
Il sera tenu informé de la survenue éventuelle d'évènements indésirables, et décidera après enquête de la poursuite ou non de l'étude.
Le comité indépendant peut être saisi par le comité de pilotage de l'étude ou par le promoteur.

Le comité indépendant sera informé par courrier électronique et par fax de chaque passage en réanimation. Tout membre du comité indépendant peut demander la réunion dudit comité pour analyser les dossiers des enfants ayant fait l'objet d'un transfert en réanimation.
Il pourra demander s'il le juge utile un moratoire sur les inclusions et une analyse intermédiaire pour s'assurer qu'il n'y a pas lieu d'arrêter prématurément l'étude, le bénéfice du traitement semblant constitué ou son caractère délétère démontré.

Les résultats des analyses intermédiaires lui seront présentées (et à lui seul), et il décidera au vu de ces résultats de la poursuite partielle (ie pour une classe d'âge particulière) ou totale de l'essai. Si au vu de l'une de ces analyses intermédiaires, il conclut à l'efficacité ou a l'effet délétère de l'intervention sur une classe d'âge ou pour tous les enfants, il pourra demander l'arrêt prématuré des inclusions.

Les modalités d'information du comité d'expert en cas d'évènement indésirable sont les suivantes : l'investigateur coordonnateur et le promoteur sont informés par l'investigateur local de la survenue d'un évènement indésirable dans un délai le plus bref possible (12 heures les jours ouvrables, 48 heures les week-end et jours fériés). L'investigateur coordonnateur transmet l'information (âge de l'enfant, type d'évènement indésirable, et lieu d'hospitalisation de l'enfant) par mail et par fax à chacun des membres du comité indépendant. Ceux-ci peuvent demander des informations complémentaires, et s'ils le jugent nécessaire, que leur soit communiqué le bras de traitement dans lequel était inclus l'enfant.

9 ASPECTS MÉDICO-LÉGAUX

9.1 Engagements de l'investigateur - Bonnes Pratiques

Des audits peuvent être décidés par le promoteur, les autorités locales ou les autorités à qui les informations concernant cette étude ont été soumises. Tous les documents en rapport avec cette étude doivent être disponibles pour une telle inspection après avis préalable.

9.1.1 Conservation de la documentation et des rapports

L'investigateur archivera et conservera pendant au moins 15 ans après la fin de l'étude les documents suivants relatifs à l'étude :

- version actualisée du protocole et des annexes ; amendements éventuels
- cahiers d'observation
- consentements éclairés des patients inclus

- correspondance relative à l'essai

Les dossiers sources des patients devront également rester disponibles pendant tout ce temps.

9.1.2 Fin prématurée de l'étude

A tout moment, le promoteur et/ou l'investigateur peuvent interrompre l'essai prématurément pour une raison médicale et/ou administrative. Dans tous les cas, l'arrêt ne pourra se faire qu'après concertation mutuelle et documentation appropriée des motifs (ex : lettre d'abandon pour l'investigateur). L'investigateur retournera alors les cahiers d'observations et toute documentation se rapportant à l'étude au promoteur.
L'investigateur principal informera le CCPPRB.

9.1.3 Monitoring

Avant de débuter l'essai, les investigateurs communiqueront à la coordination clinique les unités et les valeurs normales des paramètres biologiques et cliniques explorés.
L'investigateur faxera à la coordination clinique (numéro de télécopie : 01 45 37 40 20) les fiches d'inclusions dans les 24 heures qui suivent l'inclusion d'un patient.
L'étude sera monitorée par des visites sur site du Dr Gajdos et/ou de l'attaché de recherche clinique et des appels téléphoniques réguliers.
Un temps suffisant devra être consacré par l'investigateur à ces visites sur site. Les données des patients seront rendues disponibles pour une inspection éventuelle par les autorités compétentes ou par les représentants du promoteur. Toutefois, les patients ne seront pas identifiés par leur nom et une stricte confidentialité sera préservée à tout moment.
La vérification des données est également requise et sera faite par consultation directe des documents sources si le patient a donné son accord, dans le respect de la confidentialité.
Les principaux paramètres d'évaluation doivent être retrouvés dans les documents sources.

Le consentement éclairé de chaque patient inclus sera vérifié.
En fin d'essai, tout matériel non utilisé sera récupéré par le moniteur de l'étude.

9.2 Assurance et Contrôle de qualité

Le promoteur a souscrit pour toute la durée de l'étude, une assurance garantissant sa responsabilité civile ainsi que la responsabilité de tous les investigateurs impliqués dans la réalisation de l'étude. Il assurera également l'indemnisation intégrale des conséquences dommageables à la recherche pour la personne qui s'y prête, sauf preuve à la charge que le dommage n'est imputable à leur faute, sans toutefois que puisse être opposé le fait d'un tiers ou le retrait volontaire de la personne qui avait initialement consenti à se prêter la recherche (loi n° 88-1138, Art. L. 209-7).

9.2.1 Promoteur.

Le promoteur de l'étude est l'Assistance Publique – Hôpitaux de Paris, Délégation Régionale à la recherche clinique (DRRC), Hôpital saint Louis, 1, rue Claude Vellefaux, 75010 Paris.

9.2.2 Consentement éclairé des patients ou volontaires.

Les parents des patients seront informés par leur médecin de l'objectif, de la nature, des contraintes et des risques prévisibles de l'essai. Une notice explicative leur sera remise ainsi qu'un formulaire de consentement éclairé qu'il devra dater et signer avant de débuter l'essai.

Afin d'assurer la confidentialité médicale et la protection des données, les formulaires de consentement écrit seront conservés par l'investigateur pendant une période de quinze ans après la fin de l'essai. L'investigateur attestera dans le cahier d'observation que le consentement du patient a été obtenu en datant et en signant.

L'investigateur ne débutera aucun examen spécifiquement requis par l'essai avant d'avoir obtenu le consentement écrit des parents du patient. Les parents des patients seront informés que toutes les données de l'essai seront informatisées et conservées de façon confidentielle. Les noms des patients étant maintenus secrets, la documentation et l'évaluation des données seront identifiées uniquement par les trois premières lettres du nom de famille et les deux premières lettres du prénom de l'enfant ainsi que par le numéro individuel de patient.

9.2.3 CCPPRB.

Le protocole technique et les documents annexes ont été soumis par le coordinateur de l'étude au CCPPRB de Saint Germain-en-Laye qui a émis un avis favorable lors de sa délibération du 13 mai 2004.

La DRC déclarera l'essai à la Direction Générale de la Santé.

9.2.4 Lettre d'intention – Officialisation du début de l'étude.

On demandera le curriculum vitae de l'investigateur coordonnateur et des autres investigateurs participant à l'étude, ainsi que l'accord signé de chaque investigateur responsable d'un centre, pour sa participation à l'étude.

9.2.5 Assurance

Le promoteur a souscrit une assurance spécifique à la recherche Convention financière (Société GERLING France N° 01 / 8088731).

9.2.6 Aspects pharmaceutiques

Sans objet

9.3 Cahiers d'observation

Il permet le recueil des données à l'inclusion et du critère de jugement. Une surveillance toutes les 8 heures y est consignée.

9.4 Modifications et amendements au protocole :

Toute modification apportée au protocole après son approbation par le CCPPRB ne sera réalisée qu'exceptionnellement et fera l'objet d'un écrit qui sera signé par l'investigateur et le promoteur.
Tout amendement sera transmis par le coordonnateur de l'essai au CCPPRB, qui donnera son avis et à la DRRC qui en fera la déclaration à la Direction Générale de la Santé.

9.5 Anonymat des patients ou volontaires inclus

Toute documentation non publiée transmise à l'investigateur, est confidentielle. Ces documents ne doivent pas être dévoilés à une tierce personne sans l'accord du promoteur. La soumission de ces documents au CCPPRB est formellement autorisée. Le promoteur est libre de soumettre les données et résultats de cette étude aux gouvernements et autorités habilités.

9.6 Durée de l'étude

La durée prévue de l'étude est de trois années et demie. Si le nombre de sujets inclus dépasse le nombre de sujets nécessaires avant les trois années et demie, l'étude sera fermée dés le nombre de sujets nécessaires inclus.

9.7 Archivage des documents

Il se fera conformément à la réglementation.

10 RAPPORT ET PUBLICATION(S)

Un rapport final sera rédigé par le biostatisticien. Il inclura les tableaux des données brutes et le rapport statistique des données. Ce rapport sera soumis pour approbation et signature au principal investigateur.
L'analyse des résultats fera également l'objet de communications dans les congrès et de publications.
Le texte des publications et des communications sera discuté avec l'ensemble des investigateurs participant à l'essai. L'ordre des co-auteurs tiendra compte de la participation des différents investigateurs à l'essai (nombre de patients inclus).

11 RÉFÉRENCES BIBLIOGRAPHIQUES :

1. Simoes EA. Respiratory syncytial virus infection. Lancet 1999;354(9181):847-52.
2. Première conférence de consensus en kinésithérapie respiratoire. In: Conférence de consensus; 1994; Lyon; 1994.
3. Chalumeau M, Foix-L'Helias L, Scheinmann P, Zuani P, Gendrel D, Ducou-le-Pointe H. Rib fractures after chest physiotherapy for bronchiolitis or pneumonia in infants. Pediatr Radiol 2002;32(9):644-7.
4. Prise en charge de la bronchiolite du nourrisson : texte long. In: Conférence de consensus; 2000; Paris; 2000.
5. Prise en charge de la bronchiolite du nourrisson : texte court. In: Conférence de consensus; 2000; Paris; 2000.

12 ANNEXES

12.1 Critères d'hospitalisation.

Aspect « toxique » (altération importante de l'état général)

Survenue d'apnées, présence d'une cyanose

Fréquence respiratoire supérieure à 60/minutes

Saturation artérielle transcutanée en oxygène inférieure à 94% sous air au repos ou lors de la prise des biberons

Troubles digestifs compromettant l'hydratation, déshydratation avec perte de poids de plus de 5%

Difficultés psycho-sociales

(D'après la conférence de consensus sur la prise en charge de la bronchiolite aiguë du nourrisson (5))

12.2 Déroulement d'une séance type de kinésithérapie.

12.2.1 Les conditions pour réaliser une AFE :

- Absence de bradycardie
- Absence de balancement thoraco-abdominal de type II (inversion thoraco-abdominale)
- Absence de geignement expiratoire
- Absence de cyanose
- Saturation transcutanée en oxygène supérieure ou égale à 90% quelque soit le débit de l'oxygénothérapie
- Absence de pauses respiratoires
- Absence de trouble de conscience
- estomac vide
- Déstabilisation de l'état respiratoire au cours de la réalisation de l'AFE

12.2.2 Désinfection rhino-pharyngée

La désinfection rhino-pharyngée (DRP) n'est indiquée en début de séance que si il existe une indication posée lors de l'examen : sécrétions nasales, inspiration nasale bruyante, gêne à l'alimentation. Elle est réalisée comme indiquée en annexe 6.

12.2.3 Test de précontrainte

Il est obligatoire avant de pratiquer une AFE. Il conditionne la réalisation de celle-ci en permettant de connaître et d'évaluer les réactions de l'enfant aux contraintes mises en jeu lors de l'AFE.
Il est pratiqué sur un temps thoracique puis un temps abdominal

<u>Premier temps : temps thoracique</u>

Mise en pression thoracique expiratoire la plus lente et la plus profonde possible en observant la réaction de l'enfant: **Si l'enfant ne réagit pas à cette contrainte (absence de douleur)** la réponse négative nous assure de l'intégrité du gril costal.

<u>Deuxième temps : temps abdominal</u>

Un temps de mise en pression abdominale la plus lente et profonde possible: **une réponse négative** (**absence de douleur**) permet d'éliminer une pathologie abdominale rendant l'AFE délétère.

Une grille d'évaluation sera remplie avant chaque séance résumant les résultats des tests de contrainte.

Pression	Réaction douloureuse (0 = non / 1 = oui)		dynamique autorisée: 0 = non / 1 = oui	
**THORACIQUE**	0. ❑	1. ❑	0. ❑	1. ❑
**ABDOMINALE**	0. ❑	1. ❑	0. ❑	1. ❑

12.2.4 Description de L'augmentation du flux expiratoire (AFE)

L'AFE est un mouvement thoraco-abdominal synchrone créé par les mains du kinésithérapeute sur le temps expiratoire. Ce geste met en compression le thorax et l'abdomen pour créer un flux expiratoire passif.

<u>Positionnement de l'enfant</u> : proclive 30° à 40° sur un plan dur, thorax et abdomen nu. Aucun objet entre le dos de l'enfant et le plan sur lequel il repose.

<u>Positionnement du kinésithérapeute</u> : fente avant de la jambe du côté de la main thoracique, mains chaudes et propres dépourvues de toute parure pouvant interposer un obstacle entre la main et le grill costal lors de l'AFE.
Le geste peut commencer dès le début de l'expiration, il s'arrête lorsqu'il n'y a plus de souffle émis : cet indicateur signe la limite des contraintes maximales autorisées par la compliance thoraco-pulmonaire.

<u>Geste d'AFE</u> :
Le geste débute dés le début de l'expiration et peut aller si besoin jusqu'à la fin de l'échappement expiratoire. Il est modulable pour son efficacité (amplitude et vitesse), sans toutefois dépasser le seuil de tolérance de la structure bronchiolaire (risque de produire un collapsus bronchio-alvéolaire).
La main thoracique enveloppante sur son bord ulnaire et les quatrièmes et cinquièmes doigts, est située entre la fourchette sternale et la ligne bi-mamelonnaire. Elle va exercer une pression de haut en bas et d'avant en arrière.
La main abdominale englobe l'abdomen, elle est centrée sur l'ombilic. Elle exerce une pression de bas en haut, et d'avant en arrière.
Les axes dynamiques des deux mains sont perpendiculaires, obliques et sagittaux, et se rejoignant en T7-T8.
Les axes d'appui font 45° par rapport au plan de référence sur lequel est posé l'enfant

Critères du geste

Critère auditif : production d'une majoration sonore sur tout le temps expiratoire, du plus grave au plus aigu.

Critère plastique : absence de blocage thoracique ou de sensation de dureté importante.

La dynamisation des secrétions est attestée (passée la toux de désencombrement haut) par

- L'obtention d'une toux après 4 à 6 AFE
- L'apparition d'une majoration sonore accompagnée de bruits de secrétions (bruits mouillés opposés aux bruits secs)
- Des bruits laryngés
- Une toux grasse
- Des vibrations senties sous la main thoracique
- Une visualisation des secrétions à la base de la langue
- Une plus grande malléabilité thoracique au fil des AFE

12.2.5 Technique de Toux provoquée

Elle se pratique le plus souvent à haut volume pulmonaire sur le plateau de fin d'inspiration. Elle nécessite pour être efficace, un ensemble musculaire expiratoire efficient.

Tête maintenue en légère extension, l'action provoquée de la toux est produite dans un premier temps par le repérage de la trachée au dessus de la fourchette sternale consistant à mettre en jeu l'étirement des parois laryngées. Dans un deuxième temps, si la toux n'est pas déjà déclenchée, la pression digitale verticale de l'index ou du pouce, avec un contre appui abdominal de l'autre main afin de suppléer la tonicité des muscles abdominaux cherche à déclencher la toux.

L'existence d'un reflux gastro-oesophagien ou la présence de douleurs abdominales au test de pré-contrainte représentent des contre-indications à la réalisation du contre appui.

Contre-indications à la toux provoquée :

- ✓ Toux émétisante
- ✓ Dyspnée laryngée
- ✓ Malformation laryngée
- ✓ Défaut de déglutition (immaturité musculaire, réflexes incomplets)
- ✓ Toux d'irritation

- ✓ Toux allergique
- ✓ Toux apnéisante (coqueluche)

12.2.6 Critères d'efficacité de l'AFE

- √ Déstabilisation de l'état respiratoire au cours de la réalisation de l'AFE
- √ Absence de toux spontanée à l'AFE
- √ Diminution ou disparition de la sibilance
- √ Diminution ou disparition des ronchi
- √ Majoration sonore expiratoire claire
- √ Toux sèche

12.2.7 Séquence

1. Désinfection rhino-pharyngée si nécessaire
2. Evaluation des contraintes
3. Alternance AFE-Toux provoquée jusqu'à obtention des critères de dynamisation des secrétions

Durée de la séance (hors examen) : 10 minutes environ

Figure 1 : position des mains pour l'AFE

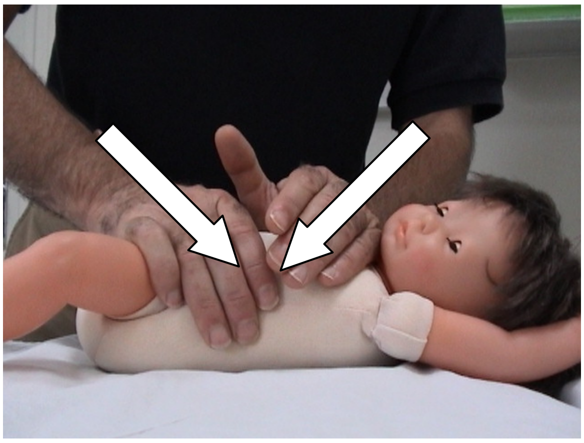

Figure 2 : geste d'AFE

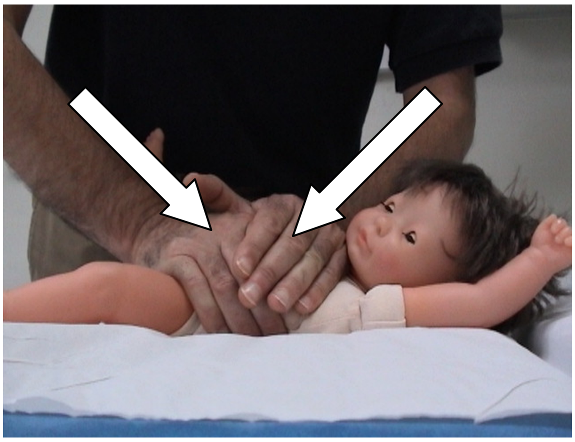

12.3 Feuille de recueil.

Fréquence Respiratoire en air ambiant (calcul sur une minute) |__||__||__| /min

Saturation artérielle transcutanée en oxygène en air ambiant |__||__||__| %

Fréquence cardiaque |__||__||__| /min

Score de lutte respiratoire ***au repos et en dehors des cris ou pleurs*** |__|

0 : pas de tirage intercostal (TIC),

1 : TIC modéré,

2 : TIC important et/ou tirage sous costal et/ou entonnoir xyphoïdien hors pectus-excavatum

3 : TIC sévère et/ou battement des ailes du nez et/ou thorax bloqué

Score de « Sibilance » ***au repos et en dehors des cris ou pleurs*** |__|

0 : pas de sibilance audible à l'oreille

1 : sibilance audible à l'oreille

12.4 Protocole standardisé d'aspiration nasale

D'après « Intérêt des aspirations des voies aériennes : Recommandations. Journées internationales en kinésithérapie respiratoire instrumentale – Lyon 16-17/11/2000

Il s'agit en fait d'une aspiration nasale et non pas naso-pharyngée. La sonde ne doit pas aller plus loin que la cavité nasale et ne doit pas pénétrer dans le pharynx. L'aspiration des sécrétions au niveau du pharynx peut être responsable de régurgitations, d'un traumatisme local et d'une bradycardie réflexe.

INTERET (Niveau III)

Prévenir la détresse respiratoire, les troubles du sommeil et de l'alimentation chez le nouveau-né et le nourrisson.

INDICATION (Niveau III)

Une aspiration nasale peut être pratiquée lorsqu'il existe une obstruction nasale gênant l'alimentation.

Le nez qui coule ne représente pas une indication.

Les aspirations sont répétées selon l'encombrement et leur fréquence doit être le plus limitée possible.

CONTRE-INDICATIONS (Niveau III)

Il ne faut pas pratiquer d'aspiration nasale en cas de prédisposition aux épistaxis : antécédents familiaux de maladie de Rendu-0sler, troubles de la coagulation.

MODALITES (Niveau III)

Cette procédure doit se faire à distance d'un repas (1h30 à 2h).

L'opérateur immobilise la tête de l'enfant.

Une sonde d'aspiration CH 6 ou 8 préalablement humidifiée est utilisée. Celle-ci est introduite dans une narine sans aspirer et en se dirigeant lentement en bas et en arrière puis l'opérateur aspire en remontant. La même manœuvre est répétée dans l'autre narine. La longueur maximale à introduire est égale à la distance qui existe entre l'aile du nez et le coin externe de l'œil.

ACCIDENTS (Niveau III)

L'aspiration nasale peut se compliquer d'un épistaxis, d'un œdème de la muqueuse et d'un trajet sous-muqueux.

12.5 Protocole standardisé de désinfection Rhino-Pharyngée

INDICATIONS

Sécrétions nasales

Inspiration nasale bruyante

Gêne à l'alimentation

Préalable à l'aspiration nasale

MODALITES : La procédure n'est réalisée qu'une fois

L'enfant est installé en latérocubitus (Position latérale de sécurité)

Une pipette de 10 ml de sérum physiologique est appliquée de manière étanche sur la narine supérieure.

Une pression lente et prolongée est appliquée sur la pipette jusqu'à écoulement du sérum physiologique par la narine inférieure.

La tête de l'enfant est retournée et la même manœuvre est réalisée sur l'autre narine.

OBJECTIF : améliorer la rhéologie des secrétions nasales.
